# New data on the distribution, biology and ecology of the longhorn beetles from the area of South and East Kazakhstan (Coleoptera, Cerambycidae)

**DOI:** 10.3897/zookeys.805.29660

**Published:** 2018-12-11

**Authors:** Lech Karpiński, Wojciech T. Szczepański, Radosław lewa, Marcin Walczak, Jacek Hilszczański, Lech Kruszelnicki, Krzysztof Łoś, Tomasz Jaworski, Marek Bidas, Grzegorz Tarwacki

**Affiliations:** 1 Museum and Institute of Zoology, Polish Academy of Sciences, Wilcza 64, 00-679 Warsaw, Poland; 2 Department of Zoology, Faculty of Biology and Environmental Protection, University of Silesia, Bankowa 9, 40-007 Katowice, Poland; 3 Department of Forest Protection, Forest Research Institute, Sękocin Stary, Braci Leśnej 3, 05-090 Raszyn, Poland; 4 Władysława Jagiełły 7c/45, 41-106 Siemianowice Śląskie, Poland; 5 Al. Chopina 102a, 05-092 Łomianki Dolne, Poland; 6 Prosta 290 D/2, 25-385 Kielce, Poland

**Keywords:** *
Anoplistes
*, Central Asia, *
Dorcadion
*, endemic species, *
Exocentrus stierlini
*, faunistics, invasive species, new records, pests, *
Psilotarsus
*, synonymy, zoogeography

## Abstract

New data on the distribution, biology and ecology of the longhorn beetles occurring in southern and eastern regions of Kazakhstan are presented together with a list of 78 species that were collected during two entomological expeditions conducted in May and June 2017. New localities of some rare taxa endemic to this region of Asia, such as *Psilotarsusbrachypterusbrachypterus* (Gebler, 1830), *Stenocorusminutus* (Gebler, 1841) and *Dorcadioncrassipescrassipes* Ballion, 1878 are given. *Exocentrusstierlini* Ganglbauer, 1883 is recorded from Kazakhstan for the first time. Moreover, the occurrence of three species: *Amarysiusduplicatus* Tsherepanov, 1980, *Rhopaloscelisunifasciatus* Blessig, 1873 and *Saperdaalberti* Plavilstshikov, 1916, which were recently found in the country, is also confirmed. Furthermore, high-quality photographs of several unique taxa, i.e. *Psilotarsusbrachypteruspubiventris* (Semenov, 1900), *Xylotrechusadspersus* (Gebler, 1830), *X.alakolensis* Karpiński & Szczepański, 2018, *Anoplistesgalusoi* (Kostin, 1974), *A.jacobsoni* Baeckmann, 1904 and *Obereakostini* Danilevsky, 1988 along with images of their habitats and feeding galleries are also presented. New localities of species considered serious pests or invasive, such as *Turaniumscabrum* (Kraatz, 1882) and *Trichoferuscampestris* (Faldermann, 1835), respectively, are also given. A new synonymy is proposed: *Cerambyxscalaris* Linnaeus, 1758 = *Cerambyxhieroglyphicus* Pallas, 1773, **syn**. **n**.

## Introduction

The cerambycid fauna of Kazakhstan is represented by ca. 272 species. Some of these, especially in genera such as *Psilotarsus*, *Dorcadion* and *Politodorcadion*, are represented by several subspecies, the total number of which extends the number of Kazakh taxa to 365 (Danilevsky 2018a).

Due to its huge area and numerous still well-preserved, heavily landlocked regions, the cerambycid fauna of Kazakhstan is quite unique. As many as 111 taxa (approx. 30%) are recognised as occurring exclusively in this country (Danilevsky 2018a). Desert, semi-desert and steppe habitats are inhabited by many endemic species, mainly from the genera *Xylotrechus* (*Kostiniclytus* subg.), *Anoplistes*, *Dorcadion*, *Politodorcadion* and *Tetrops*. Additionally, many boreal species, including some that are very rare and threatened in Europe, e.g. *Macrolepturathoracica* (Creutzer, 1799), *Lepturalianigripes* (DeGeer, 1775) and *Exocentrusstierlini* Ganglbauer, 1883, seem to be rather abundant in the foothills of the West Altai Mountains in the northeastern region of the country.

The state of the knowledge on the longhorn beetles of southern and eastern Kazakhstan as well as information about the biology and ecology of some of the species that are distributed in the region is still poor. Therefore, the present study aims to supplement the knowledge in this field. An additional goal of this work is to gather and disseminate information contained in very valuable but scattered publications, often inaccessible and usually published exclusively in Russian.

## Study area and methods

Kazakhstan is the largest landlocked country in the world with an area of 2.72 million square kilometres. Its terrain stretches west to east from the Caspian Sea to the Altai Mountains and north to south from the plains of western Siberia to the oases and deserts of Central Asia. Approximately one-third of the country’s area is occupied by the Kazakh Steppe, which is the largest dry steppe region in the world.

The climate of Kazakhstan is determined by its location in the heart of a huge continent far from the ocean, where sea-air masses do not reach. Therefore, it is of a harshly continental character with average temperatures of between -5 °C and -20 °C in January and between +18 °C and +29 °C in July, depending on the subregion. The differences in the summer-winter as well as the day-night temperatures are extremely high. In winter, the temperature may decrease to -50 °C and in summer rise up to +40 °C. There is practically no precipitation in the central part of Kazakhstan and it ranges annually from approx. 250 mm in the north to 450 mm in the mountain ranges in the south (de Beurs and Henebry 2004, MEWR 2014).

Due to its unique combination of natural complexes of steppes, deserts and mountains, which are connected via major inland water and river systems, Kazakhstan provides a wide variety of habitats and relevant types of flora. The country is also characterised by a full range of subzonal forms of steppe vegetation, deserts and mountain zones, which are typical for Central Eurasia. Approximately 75% of the area of the country is covered by arid and sub-humid lands. The lowland ecosystems in the plains consist of three main zonal types: forest- and meadow-steppes, steppes and deserts (approx. 2%, 28% and 46% of the total area, respectively). Forest-steppes are located exclusively in the north and they are largely formed by birch and aspen-birch stands. Deserts, in turn, have a high share of shrubs and semi-shrubs and they are characterised by a low species diversity, small projective cover and an absolute dominance of drought-resistant species of xerophytes and hyper-xerophytes. The mountain ecosystems cover approx. 7% of the country and they are considerably more complicated in structure and more diverse than the ecosystems of the plains. Land that is covered with forests constitutes less than 5% of the total area and these are located mainly in the northeastern part of the country (MEWR 2014).

**Map 1.**
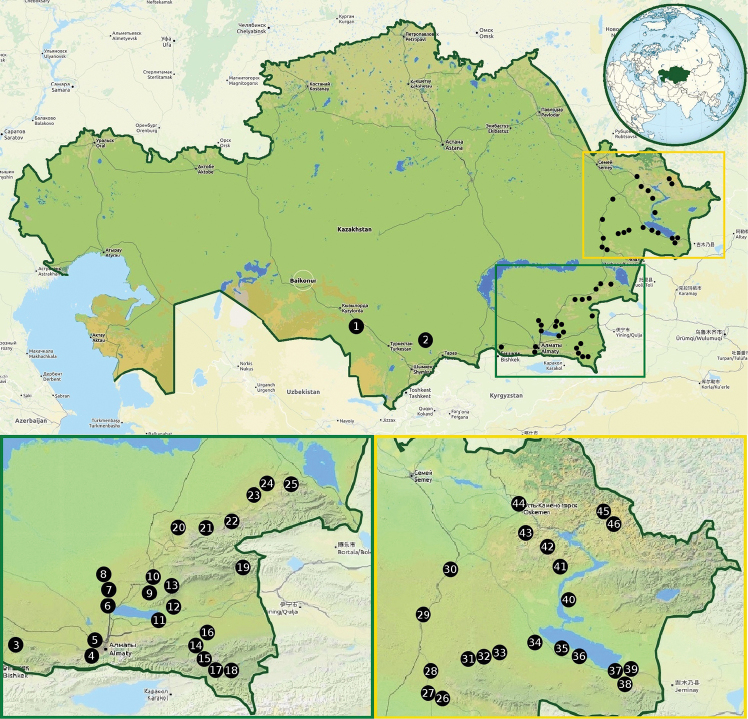
Research plots in Kazakhstan: **1** Tartogay env. (44°25'N, 66°13'E) **2** 10 km NW of Akkol (43°27'N, 70°35'E) **3** 5 km W of Kenen (43°25'N, 74°58'E) **4** 10 km S of Kaskeleng (43°05'N, 76°35'E) **5** Kaskeleng (43°12'N, 76°38'E) **6** two neighbouring localities: Kapchagay (43°52'N, 77°03'E), 8 km N of Kapchagay (43°56'N, 77°02'E) **7** two neighbouring localities: 22 km N of Kapchagay (44°05'N, 77°02'E), 26 km N of Kapchagay (44°06'N, 77°03'E) **8** 50 km N of Kapchagay (44°18'N, 76°56'E) **9** 2 km E of Arkhaly (44°10'N, 77°56'E) **10** 2 km E of Saryozek (44°22'N, 78°01'E) **11** 5 km N of Karashota (43°41'N, 78°09'E) **12** 25 km SW of Kalinino **13** Karlygash env. (44°16'N, 78°28'E) **14** 38 km SW of Szonży (43°21'N, 79°03'E) **15** four neighbouring localities: 2 km N of Kegen (43°02'N, 79°13'E), 10 km N of Kegen (43°09'N, 79°12'E and 43°07'N, 79°11'E), 15 km N of Kegen (43°09'N, 79°12'E) **16** 13 km W of Szonży (43°32'N, 79°17'E) **17** 17 km SE of Kegen (42°55'N, 79°25'E) **18** 5 km E of Saryzhaz (42°55'N, 79°40'E) **19** 7 km N of Sarymbel (44°29'N, 80°04'E) **20** two neighbouring localities: 1 km E of Tambala (45°14'N, 78°38'E), 34 km W of Kapal (45°14'N, 78°39'E) **21** three neighbouring localities: 15 km E of Kapal (45°11'N, 79°12'E), 16 km NE of Kapal (45°12'N, 79°14'E), 22 km E of Kapal (45°13'N, 79°16'E) **22** 10 km SW of Sarkan (45°21'N, 79°48'E) **23** 6 km E of Koylik (45°38'N, 80°19'E) **24** two neighbouring localities: Kabanbay (45°50'N, 80°37'E), 7 km W of Kabanbay (45°48'N, 80°31'E) **25** 10 km E of Gerasimovka (45°48'N, 80°59'E) **26** 3 km N of Taskesken (47°14'N, 80°47'E) **27** 15 km NW of Taskesken (47°18'N, 80°36'E) **28** 50 km S of Ajagöz (47°37'N, 80°38'E) **29** 48 km N of Ajagöz (48°22'N, 80°29'E) **30** two neighbouring localities: 120 km NE of Ajagöz (48°57'N, 80°55'E), 125 km NE of Ajagöz (48°57'N 80°54'E) **31** Tarbagatay env. (47°47'N, 81°17'E) **32** five neighbouring localities: 25 km E of Tarbagatay (47°46'N, 81°36'E), 27 km E of Tarbabatay (47°46'N, 81°36'E), 15 km W of Tarbagatay (47°46'N, 81°37'E), 20 km W of Tarbagatay (47°47'N, 81°42'E), 25 km W of Tarbagatay (47°50'N, 81°49'E) **33** 10 km E of Kyzyl Kesik (47°53'N, 82°06'E) **34** Zhantikei env. (48°04'N, 82°42'E) **35** 20 km NW of Tauke (47°57'N, 83°16'E) **36** 5 km SE of Kabanbay (47°49'N, 83°37'E) **37** 20 km NW of Zaysan (47°34'N, 84°39'E) **38** 12 km S of Zaysan (47°21'N, 84°51'E) **39** two neighbouring localities: 5 km NE of Zaysan (47°30'N, 84°57'E), Aynabulak (47°33'N, 85°03'E) **40** three neighbouring localities: 5 km SE of Kuygan (48°38'N 83°32'E), 8 km NW of Kurshim (48°34'N, 83°36'E), Kurshim env. (48°34'N, 83°36'E) **41** 7 km N of Samarskoje (49°05'N, 83°20'E) **42** Verkhnie Tainty env. (49°24'N, 83°03'E) **43** 10 km S of Bayash Utepov (49°35'N, 82°28'E) **44** Ust-Kamienogorsk (50°00'N, 82°33'E) **45** Putintsevo env. (49°52'N, 84°21'E) **46** Bykovo env. (49°42'N, 84°34'E and 49°39'N, 84°33'E) (OpenStreetMap contributors).

The studies that are presented were conducted in the southern and eastern regions of Kazakhstan in the mountain, desert and steppe (desert-steppe or semi-desert) ecosystems. It is worth noting that surveys were also carried out in riverside and lakeside habitats. The beetles were collected during two entomological expeditions, which were performed by two independent research teams in 2017. The first two-week-long survey, which was primarily focused on the species of the tribe Dorcadiini, was carried out by four scientists (GT, JH, RP and TJ) from the Department of Forest Protection, Forest Research Institute (Poland) and KŁ in May. The second one-month-long expedition, which consisted of three scientists (LK, MW and WTS) from the Department of Zoology, University of Silesia (Poland) and MB, took place in June. During these surveys, many sampling trips were carried out to various locations in the southern and eastern parts of Kazakhstan in the Kyzylorda (Қызылорда), South Kazakhstan (Оңтүстік Қазақстан), Jambyl (Жамбыл), Almaty (Алматы) and East Kazakhstan (Шығыс Қазақстан) Regions (облысы). The investigations were conducted in several research plots, *inter alia*, in the villages or environs of Almaty, Kalinino, Kapchagay, Kegen, Kurshim, Narynkol, Szymkent, Taldykorgan, Taraz, Tarbagatay, Tartogay, Taskesken, Ust-Kamienogorsk, Zaysan and Zyrjanowsk (Map 1). The more stationary part of our study, which was focused especially on species associated with forest stands, was carried out in several localities in the area between the villages of Putintsevo and Bykovo.

The most effective standard methods for collecting beetles, such as attracting them to artificial light sources (Fig. 15G, H), shaking them into an entomological umbrella, sweep netting, luring them into red wine/dark beer traps (Fig. 15F) and analyses of the inhabited material, were used during the field research. The beetles were studied using an Optek SZM7045-J4L and Olympus SZH10 stereo microscope at 7–140× magnifications. Photographs of the cerambycids in nature, their host plants and habitats were taken with Canon EOS 550D and Canon EOS 600D cameras. Photographs of the habitus were taken with a Canon EOS 50D digital camera equipped with a Canon MP-E 65 mm and Canon EF 100mm macro lens. The images that were produced were stacked, aligned and combined using Zerene Stacker software (www.zerenesystems.com). The geographical coordinates were read and recorded using a Garmin Oregon 550T 3-Inch Handheld GPS Navigator. For each specimen collected, the exact location (including the GPS coordinates), altitude, date and the names of the collectors are given. Additionally, information about the general distribution and biology of the species are provided.

The following abbreviations are used in the text:

**GT** Grzegorz Tarwacki

**LK** Lech Karpiński

**RP** Radosław Plewa

**JH** Jacek Hilszczański

**MB** Marek Bidas

**TJ** Tomasz Jaworski

**KŁ** Krzysztof Łoś

**MW** Marcin Walczak

**WTS** Wojciech T. Szczepański

The specimens are preserved in the entomological collections of the Museum and Institute of Zoology Polish Academy of Sciences (MIZ), the Department of Natural History of the Upper Silesian Museum in Bytom (USMB), the Department of Forest Protection of Forest Research Institute in Sękocin Stary, as well as in the collections of the authors.

This is the third in a series of papers on Cerambycidae from the area of Central-East Asia. The first one (Kadyrov et al. 2016) was devoted to the longhorn beetles of west Tajikistan and the second (Karpiński et al. 2018) concerned the cerambycids of Mongolia.

## Results

During our two expeditions, a total of 78 species (81 taxa including subspecies) belonging to four subfamilies (Prioninae, Lepturinae, Cerambycinae, Lamiinae) was recorded. They represent approx. 30% of the known Kazakh cerambycid fauna. *Exocentrusstierlini* Ganglbauer, 1883 is recorded from Kazakhstan for the first time. Moreover, the occurrence of three species: *Amarysiusduplicatus* Tsherepanov, 1980, *Rhopaloscelisunifasciatus* Blessig, 1873 and *Saperdaalberti* Plavilstshikov, 1916, which were only recently found in the country (Danilevskaya et al. 2009), is also confirmed.

The list of the recorded taxa along with the new localities, general characteristics and remarks on the biology and ecology is presented below. Descriptions of the most common species were omitted. Taxa that are endemic to Kazakhstan are indicated with an asterisk (*).

### Prioninae Latreille, 1802

#### Prionini Latreille, 1802

##### Psilotarsus brachypterus

Taxon classificationAnimaliaColeopteraCerambycidae

(Gebler, 1830)

###### Remarks.

This species is widespread in Central Asia from the southern parts of Russia through most of the territory of Kazakhstan to Uzbekistan, Kyrgyzstan and northwestern China (Danilevsky 2000). Specimens of this species reach a length of 20–40 mm in the males and 24–45 mm in the females (up to 65 mm when measured to the end of an abdomen filled with eggs). Twelve-segmented antennae occur in both sexes. The females are flightless. The larvae develop on the roots of desert trees and shrubs (Danilevsky 2014a). Kostin (1973), however, stated that *P.brachypterus* prefers shrub species, e.g. boyalych *Salsola* and teresken *Krascheninnikovia*, in contrast to *Mesoprionusangustatus* (Jakovlev, 1887) larvae, which feed on saxaul *Haloxylon*. Our own observations seem to support this thesis since we did not notice any trees or higher ligneous shrubs at the localities of both of the subspecies that were examined. There were the typical habitats of *Artemisia*- or semi-shrub/dwarf semi-shrub deserts. According to Danilevsky (2014a), the seasonal activity of adults is rather short and only lasts about two weeks; however, based on the data from the labels, it can vary greatly depending on the year – from May to the end of July. On the other hand, the daily activity presumably may differ depending on the particular population or the weather conditions.

Five subspecies are described in this species, four of which are known to occur in Kazakhstan: *Psilotarsusbrachypterusaralensis* (Danilevsky, 2000), *P.b.brachypterus* (Gebler, 1830), *P.b.hemipterus* (Motschulsky, 1845) and *P.b.pubiventris* (Semenov, 1900). The fifth subspecies – *P.b.alpherakii* (Semenov, 1900) is distributed exclusively in Xinjiang Province of China (Danilevsky 2018a). A closely related species – *Psilotarsushirticollis* Motschulsky, 1860, which is very numerous in Kazakhstan, also consists of several subspecies.

This species is highly variable in many morphological features even within the same population, however, our own observations indicated clear differences between the two taxa collected – *P.b.brachypterus* and *P.b.pubiventris*, primarily in the type of pubescence and the sculpture of the pronotum as well as in the size and corpulence of the body. Specimens of the nominotypical subspecies are significantly smaller and slender; even the females are noticeably smaller in size than the males of *P.b.pubiventris*. The pronotum in the males of the nominative subspecies is almost entirely hairless, smoother and lustrous with a sparse and fine punctuation, while it is definitely hairier and matted with coarse and dense, locally wrinkled punctuation in *P.b.pubiventris*.

##### Psilotarsus brachypterus brachypterus

Taxon classificationAnimaliaColeopteraCerambycidae

(Gebler, 1830)

Figs 1A–C , 9A–E

###### Material examined.

East Kazakhstan Region: 8 km NW of Kurshim [Күршім] (48°37'N 83°35'E), 462 m a.s.l., 17 VI 2017, 127♂♂, 6♀♀, leg. MW; 52♂♂, 5♀♀, leg. LK; 114♂♂, 7♀♀, leg. WTS; 31♂♂, 3♀♀, leg. MB.

###### Remarks.

This taxon is distributed in the easternmost part of Kazakhstan (the Irtysh River valley – from the environs of Semei to the Zaisan Depression) and northwestern China (Xinjiang and possibly the Gansu Provinces) (Danilevsky 2000, 2018a).

The nominotypical subspecies differs mainly due to the shorter lateral process of each middle antennal joint (generally much shorter than the length of joint base) and to its glabrous and shining pronotum, which is situated peripherally, and is rarely covered with a more or less dense pubescence (Danilevsky 2000).

The mass occurrence of this taxon (approx. five hundred specimens) was observed in mid-June during warm (25 °C) weather conditions in the *Artemisia*-desert habitat (Fig. 9F). This period most likely coincided with the beginning of the appearance of females (Fig. 9B) when the males (Fig. 9A, D) were about to reach maximum abundance. The first flying male was spotted immediately after our arrival at this plot (around 9 p.m.), and therefore, it is possible that this taxon begins its activity a little earlier. The number of individuals was increasing after dusk and reached its peak around midnight (Fig. 9D). At the same time, the females were found resting or moving on the ground. They did not seem to react to the light source in any way, even from very close distance. The females were much less numerous (ratio of approx. 1:20) and barely 20 specimens were collected during a few hours of searching within a radius of approx. 800 m from the light source. They appeared to stay active most of the night. Although no mating couples were spotted, a few probably still virgin females were observed resting motionlessly and attracting the males by rising and swinging with their ovipositor exposed in order to shoot out and spray a cloud of pheromones (Fig. 9C). A similar behaviour was observed by Danilevsky (2000) in the case of *Psilotarsusturkestanicus* (Semenov, 1888) in the Samarkand environs in Uzbekistan.

It is worth noting that quite a significant portion of the individuals were found dead or still alive in the webs of the Mediterranean black widow spider *Latrodectustredecimguttatus* (Rossi, 1790) (Fig. 9E). In some places, the density of these arachnids reached a few individuals per m^2^ and the specimens of *Psilotarsus* (both males and females) were the main victim of this spider species. Therefore, it seems that the hunting activity of *L.tredecimguttatus* is among the most important factors that affect the population of this beetle.

##### Psilotarsus brachypterus pubiventris

Taxon classificationAnimaliaColeopteraCerambycidae

(Semenov, 1900)

Figs 1D–F , 9G, H

###### Material examined.

Almaty Region: 28 km N of Kapchagay [Қапшағай] (44°06'N, 77°03'E), 679 m a.s.l., 27 VI 2017, several females and many dead individuals on the road and the roadside, leg. LK, MW & WTS; 26 km N of Kapchagay [Қапшағай] (44°06'N, 77°03'E), 648 m a.s.l., 27–28 VI 2017, 6♂♂, 20♀♀ (5♂♂, 7♀♀ – dead specimens), leg. LK; 2♂♂, 26♀♀, leg. MW; 1♂, 14♀♀, leg. WTS; 1♂ (dead specimen), 4♀♀, leg. MB; 22 km N of Kapchagay [Қапшағай] (44°05'N, 77°02'E), 675 m a.s.l., 28 VI 2017, many dead individuals, leg. LK, MW & WTS.

###### Remarks.

This subspecies is distributed in the southeastern region of Kazakhstan (from the Chu-Ili Mountains to about Chilik and the Dzungarian Alatau) and northern Kyrgyzstan (from the environs of Kara-Balta to Bishkek) (Danilevsky 2000).

It is characterised by larger, wider, robust body and relatively shorter antennae with shorter and thicker joint lobes (Danilevsky 2000). The imagines can be active from the first half of May to the end of July (Ishkov and Kadyrbekov 2004).

This taxon was observed at the end of June during warm (approx. 25 °C) weather conditions, after its mating period during which mostly females were still alive and the living males (Fig. 9G) constituted only a small percentage of the whole population. Also, among the females (Fig. 9H), many specimens (approx. 60%) were damaged or already dead including old body remains. Bite traces indicated that most of the specimens were killed or posthumously bitten by small mammals. In this area, any presence of *Latrodectustredecimguttatus*, which seemed to be the main natural enemy of the aforementioned subspecies, were not observed. Moreover, many specimens that were killed by cars when attempting to pass or fly over the road were also found smashed on the asphalt or in the roadside vegetation strip. All three plots from which the beetles were collected are located within close proximity to each other and represent a temperate semi-shrub/dwarf semi-shrub desert habitat (Fig. 10A). It is worth noting that on 10 June (17 days earlier), insects had been being attracted to the light at a plot located only 2 km away from this location and that not even a single male was observed neither in the night nor in the morning of the next day. This may indeed indicate a rather short, approximately two-week-long, period of the occurrence of this subspecies, which would be in line with Danilevsky’s (2014a) findings.

It is also interesting to note that this subspecies seems to stay active for most of the day. Within two different plots, the females of *P.b.pubiventris* were observed from the late evening hours through most of the night (the last active specimens were found around 3–4 a.m.) as well as in the morning of the next day (around 9 a.m.). However, although most of the males were already dead, a few living specimens were caught only in the morning despite several hours of attempting to attract them to the light on the previous evening and night on the same plot. This unusual behaviour might be related to the end of the period of the occurrence and condition of the individuals. On the other hand, according to Danilevsky (2014a), there are contradictory observations concerning the activity of beetles. In 2001, the author collected numerous males only in the morning before the dawn (from 5 to 5:30 a.m) but with no females and no more males were caught earlier or later during the day or while attempting to attract them to a light source at night. A slightly different observation was made by S.V. Murzin in 1989 who noted the maximum activity of both sexes at the sunrise. However, according to Ishkov and Kadyrbekov (2004), there were signs of nocturnal activity.

### Lepturinae Latreille, 1802

#### Lepturini Latreille, 1802

##### Anastrangalia sequensi

Taxon classificationAnimaliaColeopteraCerambycidae

(Reitter, 1898)

Fig. 2A

###### Material examined.

East Kazakhstan Region: Putintsevo [Путинцево] env. (49°52'N, 84°21'E), 472 m a.s.l., 21–23 VI 2017, 1♂, 2♀♀, leg. WTS; 3♂♂, 4♀♀, leg. MW; Bykovo [Быково] env. (49°39'N, 84°33'E), 570 m a.s.l., 24 VI 2017, 2♀♀, leg. WTS; 4♂♂, 3♀♀, leg. LK; 1♂, 1♀, leg. MW; 1♂, leg. MB.

###### Remarks.

This is a typical Siberian species. It has been widely discussed in a previous paper concerning the longhorn beetles of Mongolia (Karpiński et al. 2018). Additionally, we present an interesting melanistic form here (Fig. 2A).

The species was not included in the Kazakh fauna by Kostin (1973), who claimed that all records of this taxon for the country actually belonged to *Anastrangaliasanguinolenta* (Linnaeus, 1761) and that the record of *A.sequensi* from “Burabay” in the Kokchetav region (Kadyrbekov et al. 2003) must represent another species – most probably *Anastrangaliareyi* (Heyden, 1885) (Danilevskaya et al. 2009). The occurrence of *A.sequensi* in Kazakhstan was finally confirmed in the same locality as we present here by Danilevskaya et al. (2009).

##### Leptura annularis

Taxon classificationAnimaliaColeopteraCerambycidae

Fabricius, 1801

###### Material examined.

East Kazakhstan Region: 7 km N of Samarskoje [Самарское] (49°05'N, 83°20'E), 626 m a.s.l., 18 VI 2017, 2♂♂, leg. MW; Putintsevo [Путинцево] env. (49°52'N, 84°21'E), 472 m a.s.l., 19–23 VI 2017, 1♂, leg. LK; 1♂, leg. MW; Bykovo [Быково] env. (49°39'N, 84°33'E), 570 m a.s.l., 24 VI 2017, 1♂, leg. WTS; 2♂♂, leg. LK; 2♂♂, leg. MW; 1♂, leg. MB.

##### Leptura duodecimguttata

Taxon classificationAnimaliaColeopteraCerambycidae

Fabricius, 1801

Fig. 1G, H

###### Material examined.

East Kazakhstan Region: Putintsevo [Путинцево] env. (49°52'N, 84°21'E), 472 m a.s.l., 20 VI 2017, 1♂, 2♀♀, leg. LK; 1♀, leg. MW.

###### Remarks.

This is a typical Siberian species that is distributed in the Siberian part of Russia and Kazakhstan, Mongolia, China, Japan and the entire Korean peninsula (Cherepanov 1990a, Danilevsky 2014a). The larvae feed on the wood of different deciduous trees. The life cycle usually lasts two years. The adults appear in the late spring and can be found on flowers, mainly from the Apiaceae and Rosaceae families (Cherepanov 1990a).

A few specimens were observed on various Apiaceae flowers in a habitat of a mixed forest in the foothills of the West Altai Mountains.

##### Leptura quadrifasciata quadrifasciata

Taxon classificationAnimaliaColeopteraCerambycidae

Linnaeus, 1758

###### Material examined.

East Kazakhstan Region: 7 km N of Samarskoje [Самарское] (49°05'N, 83°20'E), 626 m a.s.l., 18 VI 2017, 1♂, leg. WTS; Putintsevo [Путинцево] env. (49°52'N, 84°21'E), 472 m a.s.l., 19–23 VI 2017, 1♀, leg. WTS; 1♂, 1♀, leg. LK; 1♂, leg. MW; 1♀, leg. MB; Bykovo [Быково] env. (49°39'N, 84°33'E), 570 m a.s.l., 24 VI 2017, 1♂, 2♀♀, leg. WTS; 1♂, leg. LK; 1♀, leg. MW.

##### Lepturalia nigripes rufipennis

Taxon classificationAnimaliaColeopteraCerambycidae

(Blessig, 1873)

Fig. 1I, J

###### Material examined.

East Kazakhstan Region: 25 km W of Tarbagatay [Тарбагатай] (47°50'N, 81°49'E), 878 m a.s.l., 16 VI 2017, 1♂, leg. MW; Putintsevo [Путинцево] env. (49°52'N, 84°21'E), 472 m a.s.l., 19–23 VI 2017, 2♂♂, leg. LK; 3♂♂, 1♀, leg. MW; 1♀, leg. MB; Bykovo [Быково] env. (49°39'N, 84°33'E), 570 m a.s.l., 24 VI 2017, 5♂♂, 1♀, leg. WTS; 2♂♂, 2♀♀, leg. LK; 2♂♂, 3♀♀, leg. MB; 2♂♂, 1♀, leg. MW.

###### Remarks.

This is a temperate Palaearctic species that is distributed from northeastern Europe to the Far East (Švácha and Danilevsky 1989, Sama 2002). The taxon was discussed in a previous paper concerning the longhorn beetles of Mongolia (Karpiński et al. 2018).

Although the nominotypical subspecies – *Lepturalianigripesnigripes* (DeGeer, 1873) is distributed in the western part of the range, both taxa can be found in Kazakhstan. A transitional zone between these two forms is situated in the Ural Mountains. According to Danilevskaya et al. (2009), both subspecies occur together in the eastern part of European Russia and in West Siberia, e.g. in the Orenburg region where several populations are known to consist of two different colour forms. However, although specimens with red elytra can be found sporadically in Western Europe, no yellow forms are known from East Siberia or further eastwards.

Our own observations indicate that this taxon is moderately frequent in the mountain and foothill zone in northeastern Kazakhstan, particularly in more afforested areas. About twenty specimens were collected on the flowers of various plants (e.g. Apiaceae, *Rosa* sp.) in habitats such as riverine bushes with *Caragana* shrubs, a mixed forest in the foothills of the West Altai Mountains and at the edge of a mountain deciduous grove that consisted mainly of *Populus*, *Betula* and *Salix* (Fig. 15C). Birches occurred in all of these sites.

##### Lepturobosca virens

Taxon classificationAnimaliaColeopteraCerambycidae

(Linnaeus, 1758)

###### Material examined.

East Kazakhstan Region: 7 km N of Samarskoje [Самарское] (49°05'N, 83°20'E), 626 m a.s.l., 18 VI 2017, 1♂, leg. WTS; 1♂, leg. MW; Putintsevo [Путинцево] env. (49°52'N, 84°21'E), 472 m a.s.l., 19–23 VI 2017, 1♀, leg. WTS; Bykovo [Быково] env. (49°39'N, 84°33'E), 570 m a.s.l., 24 VI 2017, 1♀, leg. LK.

##### Macroleptura thoracica

Taxon classificationAnimaliaColeopteraCerambycidae

(Creutzer, 1799)

###### Material examined.

Putintsevo [Путинцево] env. (49°52'N, 84°21'E), 472 m a.s.l., 24 VI 2017, 1♀, leg. MW; 1♀, leg. WTS.

###### Remarks.

This is a typical Siberian species. It was discussed in a previous paper concerning the longhorn beetles of Mongolia (Karpiński et al. 2018).

Several individuals of *M.thoracica* were collected on dead birch trunks in this locality in June 2005 (Danilevskaya et al. 2009).

In our research, two specimens were collected in the habitat of a rather old *Populus* forest (Fig. 15F) that extends along the Khamir River in the foothills of the West Altai Mountains. The first one was caught in flight, whereas the second was beaten down from a trunk of a dead willow *Salix* sp.

##### Oedecnema gebleri

Taxon classificationAnimaliaColeopteraCerambycidae

Ganglbauer, 1889

###### Material examined.

Putintsevo [Путинцево] env. (49°52'N, 84°21'E), 472 m a.s.l., 22 VI 2017, 1♀, leg. LK.

###### Remarks.

This is a typical Siberian species that is distributed from Eastern Europe (Ukraine and European Russia) to the Pacific Ocean. *Oedecnemagebleri* is a polyphagous cerambycid that develops in the basal zones and in the stumps of different deciduous and coniferous trees and then pupates in the soil. The imagines can be found on flowers from the end of May to August (Švácha and Danilevsky 1989, Cherepanov 1990a).

Several specimens were collected on flowers in this locality in June 2005 (Danilevskaya et al. 2009).

Only a single female was observed in the habitat of a rather old *Populus* forest that extends along the Khamir River in the foothills of the West Altai Mountains.

##### Pachytodes erraticus

Taxon classificationAnimaliaColeopteraCerambycidae

(Dalman, 1817)

###### Material examined.

East Kazakhstan Region: 7 km N of Samarskoje [Самарское] (49°05'N, 83°20'E), 626 m a.s.l., 18 VI 2017, 5♂♂, 3♀♀, leg. WTS; 5♂♂, 4♀♀, leg. LK; 4♂♂, 3♀♀, leg. MW; Putintsevo [Путинцево] env. (49°52'N, 84°21'E), 472 m a.s.l., 19 VI 2017, 1♂, leg. WTS; 3♂♂, 1♀, leg. LK; 1♂, leg. MW; Bykovo [Быково] env. (49°39'N, 84°33'E), 570 m a.s.l., 24 VI 2017, 2♂♂, leg. WTS; 10 km S of Bayash Utepov [Баяш Утепов] (49°35'N, 82°28'E), 508 m a.s.l., 25 VI 2017, 1♀, leg. WTS; 1♀, leg. LK.

###### Remarks.

*Pachytodeserraticus* is a typical Palaearctic species that is distributed from Spain to East Siberia. It develops under ground in the rotten roots of different deciduous tree species. The pupation occurs in the soil (Danilevskaya et al. 2009).

This is one of the most common species in the zone of mountains and foothills in northeastern Kazakhstan. We observed this species frequently and rather numerously on the flowers of various Apiaceae species in different habitats from the roadside vegetation strips in the mountain steppe region through river canyons with *Caragana* and *Lonicera* (Fig. 15A) to a riverine and mountain mixed forests mainly with *Betula*, *Populus*, *Salix* and *Picea*.

##### Pseudovadonia livida bicarinata

Taxon classificationAnimaliaColeopteraCerambycidae

(N. Arnold, 1869)

###### Material examined.

East Kazakhstan Region: 3 km N of Taskesken [Таскескен] (47°14'N, 80°47'E), 581 m a.s.l., 14 VI 2017, 3♀♀, leg. WTS; 1♂, 2♀♀, leg. LK; 2♂♂, 2♀♀, leg. MW; 15 km W of Tarbagatay [Тарбагатай] (47°46'N, 81°37'E), 1072 m a.s.l., 15 VI 2017, 5♂♂, leg. WTS; Kurshim [Күршім] env. (48°34'N, 83°36'E), 406 m a.s.l., 17 VI 2017, 1♂, 1♀, leg. MB; 5 km SE of Kuygan [Құйған] (48°38'N 83°32'E), 439 m a.s.l., 18 VI 2017, 1♂, 1♀, leg. LK; 7 km N of Samarskoje [Самарское] (49°05'N, 83°20'E), 626 m a.s.l., 18 VI 2017, 1♀, leg. WTS; Putintsevo [Путинцево] env. (49°52'N, 84°21'E), 472 m a.s.l., 19 VI 2017, 2♀♀, leg. WTS; 1♂, 1♀, leg. MW; Bykovo [Быково] env. (49°39'N, 84°33'E), 570 m a.s.l., 24 VI 2017, 1♂, 1♀, leg. MW.

###### Remarks.

This is the easternmost subspecies that is distributed from Eastern Europe to East Siberia and China (Danilevsky 2018a).

This taxon was recorded from the Putintsevo environs by Danilevskaya et al. (2009) under an incorrect name, *Pseudovadonialividapecta* (K. Daniel & J. Daniel, 1891). This subspecies seems to be endemic to Italy (Danilevsky 2018a).

##### Stenurella bifasciata bifasciata

Taxon classificationAnimaliaColeopteraCerambycidae

(O. F. Müller, 1776)

###### Material examined.

East Kazakhstan Region: 3 km N of Taskesken [Таскескен] (47°14'N, 80°47'E), 581 m a.s.l., 14 VI 2017, 1♂, leg. WTS; 1♀, leg. LK; 1♂, 1♀, leg. MW; 15 km W of Tarbagatay [Тарбагатай] (47°46'N, 81°37'E), 1072 m a.s.l., 15 VI 2017, 1♂, 1♀ leg. WTS; 2♂♂, 1♀, leg. MW; 7 km N of Samarskoje [Самарское] (49°05'N, 83°20'E), 626 m a.s.l., 18 VI 2017, 1♂, leg. WTS; 3♂♂, 2♀♀, leg. LK; Putintsevo [Путинцево] env. (49°52'N, 84°21'E), 472 m a.s.l., 22 VI 2017, 1♀, leg. MW; 10 km S of Bayash Utepov [Баяш Утепов] (49°35'N, 82°28'E), 508 m a.s.l., 25 VI 2017, 1♂, 3♀♀, leg. LK.

###### Remarks.

The nominotypical subspecies is distributed from Central Europe to East Siberia and China (Danilevsky 2018a).

According to some authors (e.g. Bense 1995, Sama 2002), although the biology of *S.bifasciata* is inadequately known, its larvae develop in both deciduous (*Ulmus*, *Quercus*, *Salix*, *Rosa*, *Spartium*, *Ficus*) and coniferous (*Pinus*) trees. Although our own observations from Central Europe indicate its close relationship with pines *Pinus* spp., in Kazakhstan, we also collected this species in completely treeless areas in which the only suitable host plant was the rose *Rosa* sp. We did not observe any morphological differences between the specimens from the semi-steppe habitats, which are apparently associated with *Rosa*, or from the Kazakh mountain forests having a share of *Pinus*, or from Poland.

##### Stenurella melanura melanura

Taxon classificationAnimaliaColeopteraCerambycidae

(Linnaeus, 1758)

###### Material examined.

East Kazakhstan Region: 7 km N of Samarskoje [Самарское] (49°05'N, 83°20'E), 626 m a.s.l., 18 VI 2017, 2♂♂, leg. WTS; 2♂♂, 2♀♀, leg. MW; Putintsevo [Путинцево] env. (49°52'N, 84°21'E), 472 m a.s.l., 19–23 VI 2017, 2♂♂, 1♀, leg. WTS; 2♀♀, leg. MW; Bykovo [Быково] env. (49°39'N, 84°33'E), 570 m a.s.l., 24 VI 2017, 1♂, 1♀, leg. WTS; 1♀, leg. LK.

##### Strangalia attenuata

Taxon classificationAnimaliaColeopteraCerambycidae

(Linnaeus, 1758)

###### Material examined.

7 km N of Samarskoje [Самарское] (49°05'N, 83°20'E), 626 m a.s.l., 18 VI 2017, 2♂♂, 2♀♀, leg. WTS; 1♂, 1♀, leg. LK; 1♂, leg. MW; Putintsevo [Путинцево] env. (49°52'N, 84°21'E), 472 m a.s.l., 20 VI 2017, 1♂, 1♀, leg. WTS; 1♂, 3♀♀, leg. LK; 1♂, 1♀, leg. MW; Bykovo [Быково] env. (49°39'N, 84°33'E), 570 m a.s.l., 24 VI 2017, 1♂, leg. LK; 1♂, 1♀, leg. MW; 10 km S of Bayash Utepov [Баяш Утепов] (49°35'N, 82°28'E), 508 m a.s.l., 25 VI 2017, 1♂, leg. WTS.

#### Rhagiini Kirby, 1837

##### Brachyta interrogationis russica

Taxon classificationAnimaliaColeopteraCerambycidae

(Herbst, 1784)

Fig. 2B

###### Material examined.

Bykovo [Быково] env. (49°39'N, 84°33'E), 571 m a.s.l., 21 VI 2017, 1♀, leg. LK.

###### Remarks.

This is a typical Palaearctic species that is distributed from Spain to the Russian Far East, Korea and China. *Brachytainterrogationis* is a very variable taxon with twelve described subspecies. Each local population is characterised by unique proportions of certain colour forms. The *russica* ssp. is known to occur in European Russia (except for the northern Urals), West Siberia (including Altai) and Kazakhstan, and it is the only subspecies that has been recorded from Kazakhstan (Lazarev 2016, Danilevsky 2018a). In Siberia, the larvae of this species have usually been observed in the roots of living *Paeonia* but also those of *Euphorbia* and *Radiola*, as well as *Trollius* in laboratory conditions (Danilevskaya et al. 2009). Pupation occurs in the soil. The adults can be found on the flowers of various plants from May to the turn of July and August. The species is most numerous in the foothill and mountain regions of the forest and forest-steppe zones (Cherepanov 1990a).

Several individuals of this taxon were collected on *Paeonia* in the area of Putinzevo and on *Ranunculus* in the Sibinka River valley in June 2005 (Danilevskaya et al. 2009), however no subspecies was specified.

A single female was collected on the stem of a herbaceous plant species at the edge of a mountain deciduous grove that consisted mainly of *Populus*, *Betula* and *Salix* (Fig. 15C).

##### Dinoptera collaris

Taxon classificationAnimaliaColeopteraCerambycidae

(Linnaeus, 1758)

###### Material examined.

East Kazakhstan Region: 3 km N of Taskesken [Таскескен] (47°14'N, 80°47'E), 581 m a.s.l., 14 VI 2017, 1♂, leg. MW; 7 km N of Samarskoje [Самарское] (49°05'N, 83°20'E), 626 m a.s.l., 18 VI 2017, 1♀, leg. MW; Putintsevo [Путинцево] env. (49°52'N, 84°21'E), 472 m a.s.l., 21 VI 2017, 4♀♀, leg. WTS; 3♂♂, 3♀♀, leg. LK; Bykovo [Быково] env. (49°39'N, 84°33'E), 570 m a.s.l., 24 VI 2017, 1♀, leg. MW.

##### Stenocorus minutus

Taxon classificationAnimaliaColeopteraCerambycidae

(Gebler, 1841)

Fig. 2C, D

###### Material examined.

East Kazakhstan Region: 10 km S of Bayash Utepov [Баяш Утепов] (49°35'N, 82°28'E), 508 m a.s.l., 25 VI 2017, 4♂♂, 2♀♀, leg. LK; 1♂, 1♀, leg. WTS; 1♂, leg. MW; 1♂, leg. MB.

###### Remarks.

*Stenocorusminutus* is a rare species that is distributed mainly in eastern Kazakhstan and reaches northwestern China and western Mongolia through the Irtysh River valley and the Tarbagatay Mountain range (Danilevsky 2014a, 2018a). While the biology of the preimaginal stages remains unknown, according to Danilevsky (2014a), larvae of this species undoubtedly descend under ground where they feed on the roots of various woody plants. Kadyrbekov and Childebaev (2007) claimed that the larvae develop in dead deciduous trees. The latter authors found adults on the flowers of various plant species, including *Euphorbialamprocarpa*, in the second half of June. On the other hand, the species was quite numerously observed in treeless xerothermic habitats that had a significant number of shrubs (Danilevsky 2014a).

For a long time, *Stenocorusminutus* was known in Kazakhstan only from the upper ranges of the Saur and Tarbagatay Mountains (Kostin 1973). It was also mentioned as occurring in southeastern Kazakhstan (along Lake Zaysan) by Cherepanov (1990a) under its outdated name *Stenocorustataricus* (Gebler, 1841); however, the author had never observed this species in nature. Recently, it was found more frequently in the riparian forests of the Tentek River valley (Kadyrbekov and Childebaev 2007).

About ten specimens were collected on the leaves and stems of *Caragana* and *Lonicera* shrubs overgrowing the stony hills in the Sibinka River valley (Fig. 15B). A female was observed feeding on juice leaking from a damaged *Caragana* stem together with a few individuals of *Protaetia* spp. That observation and the fact that most of the specimens (also couples together) were found on *Caragana* may indicate an association of *Stenocorusminutus* with this plant genus, especially since not a single specimen has been recorded by any of the expeditions in a rather well-investigated area of Putintsevo, that is located approx. 120 km eastward, in which many deciduous trees (e.g. *Betula*, *Padus*, *Populus*, *Salix*) and shrubs (e.g. *Lonicera*, *Rosa*, *Spiraea*, *Viburnum*) occur with the exception of *Caragana*. It is also worth noting that this rare but rather large and easily spotted species has not been found in presented locality by M. Danilevsky’s research team in mid-June of 2005, which may indicate the beginning of the appearance of beetles in the second half of June when the adults of their sympatric species *Obereakostini* end their activity.

### Cerambycinae Latreille, 1802

#### Callidiini Kirby, 1837

##### Turanium scabrum

Taxon classificationAnimaliaColeopteraCerambycidae

(Kraatz, 1882)

Fig. 2E–G

###### Material examined.

Almaty Region: 1 km E of Tambala [Тамбала] (45°14'N, 78°38'E), 663 m a.s.l., 3 V 2017 (6 V 2017 ex cult.) 2♂♂, 2♀♀, from *Elaeagnusangustifolia*, leg. JH; 13 km W of Szonży [Шонжы] (43°32'N, 79°17'E), 731 m a.s.l., 12 V 2017 (I 2018 ex cult.) 1♀, from *Elaeagnusangustifolia*, leg. KL; 5 km N of Karashota [Каражота] (43°41'N, 78°09'E), 492 m a.s.l., 3 VI 2017, 1♂, 2♀♀, leg. WTS; 1♂, 1♀, leg. LK; 5♂♂, 3♀♀, leg. MW.

###### Remarks.

The species is distributed from the southern region of European Russia through the countries of Central Asia to West Siberia and China (Danilevsky 2018a). The polyphagous larvae develop in various deciduous trees and shrubs (e.g. *Elaeagnus*, *Populus*, *Malus*, *Rosa*, *Halimodendron* and *Tamarix*). The adults are active from April to July (Danilevsky 2001b, Ishkov and Kadyrbekov 2004).

*Turaniumscabrum* inhabits almost all of the territory of Kazakhstan (excluding northern and northeastern regions) (Danilevsky 2001b) and it is considered a serious pest, especially of *Elaeagnusangustifolia* and *Populusdiversifolia* (Borissova 2018).

The individuals were observed during a hot (30 °C) and sunny afternoon on a flood barrier formed from old branches and boughs primarily of oleasters *Elaeagnus* (Fig. 10B). The beetles were flying to accumulated wood where they were mating. The species was observed sympatrically with *Chlorophoruselaeagni* there. Several specimens were additionally rared from the dry wood of *Elaeagnusangustifolia*.

#### Clytini Mulsant, 1839

##### Chlorophorus elaeagni

Taxon classificationAnimaliaColeopteraCerambycidae

Plavilstshikov, 1956

###### Material examined.

Almaty Region: 13 km W of Szonży [Шонжы] (43°32'N, 79°17'E), 731 m a.s.l., 12 V 2017 (I 2018 ex cult.) 1♀, from *Elaeagnusangustifolia*, leg. KL; 5 km N of Karashota [Каражота] (43°41'N, 78°09'E), 492 m a.s.l., 3 VI 2017, 3♂♂, leg. LK; 1♂, leg. WTS; Kyzylorda Region: Tartogay env. [Тартогай] (44°25'N, 66°13'E), 135 m a.s.l., 7 VI 2017, 3♂♂, leg. WTS; 2♂♂, leg. LK; 3♂♂, leg. MW.

###### Remarks.

This species is distributed from the Caucasus to Central Asia (Danilevsky 2018a). It was discussed in a previous paper concerning the longhorn beetles of Tajikistan (Kadyrov et al. 2016).

*Chlorophoruselaeagni* is a rather common species in southern Kazakhstan, where it mainly occurs in tugay habitats. Although the specimens were collected on sites that were located near river banks in rather different habitats, there was always a large share of *Elaeagnusangustifolia*, which seems to be the main host plant for this species. In the environs of Karashota, during a hot (30 °C) and sunny afternoon, the specimens were observed on a flood barrier formed from old branches and boughs primarily of oleasters *Elaeagnus* (Fig. 10B). Single males were flying to accumulated wood from time to time and started to actively run after landing. This period seemed to be the beginning of the occurrence of this species in nature. On this plot, *Ch.elaeagni* occurred sympatrically with *Turaniumscabrum*, which was swarming at that time. Additionally, on a bank of the Syr Darya River in a locality near Tartogay, several males were observed during a scorching (35 °C) day on blossoming tamarisks *Tamarix* (Fig. 10C) in the same habitat as *Anoplistesjacobsoni* (Fig. 11D) at the peak of its occurrence.

##### Cyrtoclytus capra

Taxon classificationAnimaliaColeopteraCerambycidae

(Germar, 1824)

###### Material examined.

East Kazakhstan Region: Putintsevo [Путинцево] env. (49°52'N, 84°21'E), 472 m a.s.l., 19–23 VI 2017, 6♂♂, 3♀♀, leg. LK; 3♂♂, 2♀♀, leg. WTS; 5♂♂, 3♀♀, leg. MW; 3♂♂, 2♀♀, leg. MB; Bykovo [Быково] env. (49°39'N, 84°33'E), 570 m a.s.l., 24 VI 2017, 2♂♂, 1♀, leg. LK; 1♂, 1♀, leg. WTS; 1♂, 1♀, leg. MW; 10 km S of Bayash Utepov [Баяш Утепов] (49°35'N, 82°28'E), 508 m a.s.l., 25 VI 2017, 1♂, 1♀, leg. WTS; 1♀, leg. MB.

###### Remarks.

The species is distributed from the northern and central parts of Europe through Siberia, including the northern regions of Kazakhstan, Mongolia and China, to the Far East and the Korean Peninsula (Danilevsky 2018a). While *Cyrtoclytuscapra* is a rather rare and sporadic species in West Europe, where the range of its host plants seems to be very narrow (*Acer*, *Alnus*), it is very numerous in Siberia, where it develops in many deciduous trees, e.g. *Betula, Quercus* and *Sorbus* but also in *Euonymus, Vitis* and *Aralia* (Danilevskaya et al. 2009). It primarily inhabits deciduous and mixed forests. The adults are active from June to August (Švácha and Danilevsky 1988).

Numerous specimens were observed throughout the day on the flowers of various herbaceous plants in rather different habitats, such as a mountain riverine forest dominated by *Salix, Populus* and *Betula*, mountain deciduous forests (Fig. 15F) or river canyon hills with *Caragana* and *Lonicera* shrubs (Fig. 15A).

##### Echinocerus floralis

Taxon classificationAnimaliaColeopteraCerambycidae

(Pallas, 1773)

###### Material examined.

East Kazakhstan Region: 3 km N of Taskesken [Таскескен] (47°14'N, 80°47'E), 581 m a.s.l., 14 VI 2017, 1♂, leg. WTS; 3♂♂, 1♀, leg. LK; 15 km NW of Taskesken [Таскескен] (47°18'N, 80°36'E), 15 VI 2017, 627 m a.s.l., 2♂♂, leg. WTS; 1♂, 1♀, leg. LK; 2♂♂, 2♀♀, leg. MW; 1♂, leg. MB; 15 km W of Tarbagatay [Тарбагатай] (47°46'N, 81°37'E), 1072 m a.s.l., 15 VI 2017, 1♀, leg. WTS; Tarbagatay [Тарбагатай] env. (47°47'N, 81°17'E), 964 m a.s.l., 15 VI 2017, 1♂, leg. WTS; 25 km W of Tarbagatay [Тарбагатай] (47°50'N, 81°49'E), 878 m a.s.l., 16 VI 2017, 1♀, leg. LK; Kurshim [Күршім] env. (48°34'N, 83°36'E), 406 m a.s.l., 17 VI 2017, 3♂♂, leg. WTS; 10 km S of Bayash Utepov [Баяш Утепов] (49°35'N, 82°28'E), 508 m a.s.l., 25 VI 2017, 3♂♂, leg. WTS; 2♀♀, leg. LK; 1♂, leg. MW.

###### Remarks.

This widespread thermophilous species is distributed from Europe throughout Asia Minor, the Caucasus, Transcaucasia, the Middle East and Central Asia to the Xinjiang region in China (Danilevsky 2018a). The larvae develop in the stems and roots of various herbaceous plants (e.g. *Medicago*, *Onobrychis*, *Amaranthus* and *Cornelia*, *Melilotus*) (Cherepanov 1990b). The adults are active in June and July, when they can be frequently observed visiting flowers of various plant species (Sama 2002).

The imagines were found in many rather dry or ruderal habitats, such as the roadside vegetation strips (Fig. 12C), hills with *Rosa* and canyons at riverbanks with *Caragana* and *Lonicera* (Fig. 15A).

##### Rhaphuma gracilipes

Taxon classificationAnimaliaColeopteraCerambycidae

(Faldermann, 1835)

Fig. 4A, B

###### Material examined.

East Kazakhstan Region: Putintsevo [Путинцево] env. (49°52'N, 84°21'E), 472 m a.s.l., 19–23 VI 2017, 1♂, 1♀, leg. WTS; 2♂♂, 2♀♀, leg. LK; 1♂, 1♀, leg. MB; 3♂♂ (1♂ – red wine trap), 2♀♀, leg. MW.

###### Remarks.

This is an east-Palaearctic species that is distributed from Eastern Europe, where is rather rare, through Siberia, including the northern regions of Kazakhstan, Mongolia and China, to Sakhalin and Japan (Kurzawa 2012, Danilevsky 2018a). While the larvae usually develop in freshly dead twigs and stems under bark, then in the wood of various deciduous plant species, mainly in *Betula*, *Acer*, *Quercus*, *Tilia* and *Ulmus*, it is also known from *Aralia*, *Vitis*, *Spiraea*, *Syringa*, *Euonymus*, *Daphne* and *Micromeles* (Danilevskaya et al. 2009). The adults are active from June to September (Sama 2002).

The species was recorded from Kazakhstan for the first time by Kostin (1973). Some specimens were also collected in the Putintsevo environs in June 2005 by Danilevskaya et al. (2009).

Several imagines were collected on the flowers of Apiaceae in a mountain deciduous forest dominated by *Populus* and *Betula* (Fig. 15F). A single male was additionally lured into a red wine trap.

##### Xylotrechus adspersus

Taxon classificationAnimaliaColeopteraCerambycidae

(Gebler, 1830)

Fig. 2H

###### Material examined.

East Kazakhstan Region: Bykovo [Быково] env. (49°42'N 84°34'E), 477 m a.s.l., 21 VI 2017, 1♀, leg. LK.

###### Remarks.

*Xylotrechusadspersus* is distributed from Altai to Sakhalin and Japan and from Yakutia to northern China and the northern part of the Korean Peninsula (Cherepanov 1990b, Danilevsky 2018a). The species is ecologically associated with willows and *Choseniaarbutifolia*. After mating, the females lay their eggs in living twigs. The larvae initially live under the bark and then in wood where they pupate after about two years. The imagines usually emerge from June to July (Cherepanov 1990b, Danilevskaya et al. 2009).

Several specimens were collected in the Putintsevo environs in June 2005 by Danilevskaya et al. (2009).

A single, probably freshly emerged, female was observed sitting motionlessly on a willow branch next to its emergence hole in an enclave of willows located next to a river (Fig. 12A). Moreover, several fresh larval feeding galleries of this species (Fig. 12B) were found in willow branches that were still alive, together with a single early instar larva under bark. Additionally, a few imagines of an unidentified parasitic Hymenoptera species were found in tunnels.

##### Xylotrechus alakolensis

Taxon classificationAnimaliaColeopteraCerambycidae

Karpiński & Szczepański, 2018 *

Fig. 2I

###### Material examined.

East Kazakhstan Region: 15 km NW of Taskesken [Таскескен] (47°18'N, 80°36'E), 15 VI 2017, 627 m a.s.l., 1♂, leg. WTS.

###### Remarks.

This newly described species was identified based on the specimen presented herein (Fig. 2I). Although only the holotype is known as yet, the range of this taxon is most likely limited to the eastern part of Kazakhstan. More information is provided in Karpiński and Szczepański (2018).

A single male was collected within a very rich roadside vegetation strip in a steppe-like habitat (Fig. 12C) using the sweep-netting method.

##### Xylotrechus capricornus

Taxon classificationAnimaliaColeopteraCerambycidae

(Gebler, 1830)

Fig. 2K, L

###### Material examined.

East Kazakhstan Region: Bykovo [Быково] env. (49°39'N, 84°33'E), 570 m a.s.l., 21 VI 2017, 1♂, 1♀, leg. LK.

###### Remarks.

*Xylotrechuscapricornus* is a rare species that is distributed from Central Europe (Austria and the Czech Republic) to West Siberia (Sláma and Gutowski 1997, Danilevsky 2018a). It is probably monophagous on birch *Betula* spp. The females are very exacting in regard to the required health condition of a host plant. Although trees are occupied infrequently, many of them, especially those that are completely exposed, can be totally inhabited by numerous specimens on almost the entire surface of a trunk. The larvae first feed under the bark and then in the wood of trunks that have recently died. Pupation occurs deep in the wood. The life cycle of this species usually lasts two years, but can be extended up to three years. A situation in which two generations develop in the same host is very unusual. While the adults are active from the end of June to mid-August, imagines are rarely observed in nature – the beetles appear only on the hottest days and they usually disappear immediately after the sun sets behind the clouds or when the weather becomes windy or cold. Only a small part of a whole population can be spotted outside of the wood (Sláma and Gutowski 1997).

Until recently, the species was known from Kazakhstan only based on a single specimen that was found near Karkaralinsk (Kostin 1973). Then, in June 2005, several specimens were collected on birch bark in the Putintsevo environs by Danilevskaya et al. (2009). That was the first record in NE Kazakhstan.

In our research, a pair of *X.capricornus* was collected at the edge of a mountain deciduous grove that consisted mainly of *Populus*, *Betula* and *Salix* (Fig. 15C). Around noon, during hot weather, the beetles were copulating on birch trunks that were lying on a sun-exposed site. Our finding confirms the presence of this species in Kazakhstan.

##### Xylotrechus hircus

Taxon classificationAnimaliaColeopteraCerambycidae

(Gebler, 1825)

Fig. 2J

###### Material examined.

**Material examined.** East Kazakhstan Region: 20 km NW of Tauke [Тауқе] (47°57'N, 83°16'E), 407 m a.s.l., 6 V 2017 (VI 2017 ex cult.) 4♂♂, 2♀♀, from *Betula* sp., leg. JH.

###### Remarks.

Although the species originally occurred exclusively in Northern Asia from Altai to Japan (Cherepanov 1990b, Danilevsky 2018a), it was recently accidentally introduced into North America (e.g. LaBonte et al. 2005), where it is considered an invasive species. It was widely discussed in a previous paper concerning the longhorn beetles of Mongolia (Karpiński et al. 2018).

Several specimens were reared from birch wood *Betula* sp. collected in the hilly grove (Fig. 12D).

##### Xylotrechus rusticus

Taxon classificationAnimaliaColeopteraCerambycidae

(Linnaeus, 1758)

###### Material examined.

East Kazakhstan Region: Putintsevo [Путинцево] env. (49°52'N, 84°21'E), 472 m a.s.l., 19–23 VI 2017, 2♂♂, 1♀, leg. WTS; 1♂, leg. LK; 1♂, leg. MW; Bykovo [Быково] env. (49°39'N, 84°33'E), 570 m a.s.l., 24 VI 2017, 1♂, 1♀, leg. WTS; 2♀♀, leg. LK; 1♂, 2♀♀, leg. MW.

#### Hesperophanini Mulsant, 1839

##### Trichoferus campestris

Taxon classificationAnimaliaColeopteraCerambycidae

(Faldermann, 1835)

###### Material examined.

Almaty Region: 2 km E of Saryozek [Сарыөзек] (44°22'N, 78°01'E), 875 m a.s.l., 02 VI 2017 (23 VI 2017, ex cult.) 1♂, 1♀, from *Ulmus* sp., leg. MW & LK; East Kazakhstan Region: Ust-Kamienogorsk [Өскемен] (50°00'N, 82°33'E), 302 m a.s.l., 19 VI 2017, 2♂♂, 1♀, leg. MW.

###### Remarks.

*Trichoferuscampestris* is originally native to the southeastern Palaearctic region; however, it is now considered an invasive species that has rapidly increased its range in recent years (e.g. Grebennikov et al. 2010, Dascălu et al. 2013). It was discussed in a previous paper concerning the longhorn beetles of Tajikistan (Kadyrov et al. 2016).

Two specimens were reared from thick elm branches *Ulmus* sp. Additionally, a few imagines were attracted to artificial light sources during a warm night in a habitat of a city park.

#### Molorchini Gistel, 1848

##### Molorchus schmidti

Taxon classificationAnimaliaColeopteraCerambycidae

Ganglbauer, 1883

Fig. 4C

###### Material examined.

East Kazakhstan Region: Aynabulak [Aынабyлaқ] ad Zaysan [Зайсан] (47°33'N, 85°03'E), 508 m a.s.l., 7 V 2017 (25 V 2017, ex cult.) 2♂♂, 2♀♀, from *Elaeagnusangustifolia*, leg. RP; Almaty Region: 13 km W of Szonży [Шонжы] (43°32'N, 79°17'E), 731 m a.s.l., 12 V 2017 (XI 2017, ex cult.) 1♀, from *E.angustifolia*, leg. KL.

###### Remarks.

This species is distributed from Eastern Europe to Central Asia (Sama 2002, Danilevsky 2018a). The larvae develop in various deciduous trees and shrubs, e.g. *Salix*, *Elaeagnus*, *Cerasus*, *Populus*, *Malus*, *Prunus* (Sama 2002). In Kazakhstan, it also develops in wood of plants such as *Halimodendron*, *Hippophae* and *Rosa* (Ishkov and Kadyrbekov 2004). The imagines are active from the second half of April to the end of June and can be found on their host plants (Sama 2002).

Several specimens were reared from the twigs of *Elaeagnusangustifolia*.

#### Obriini Mulsant, 1839

##### Obrium cantharinum cantharinum

Taxon classificationAnimaliaColeopteraCerambycidae

(Linnaeus, 1767)

###### Material examined.

East Kazakhstan Region: Bykovo [Быково] env. (49°39'N, 84°33'E), 570 m a.s.l., 21 VI 2017, 1♀, leg. MW.

###### Remarks.

This is a widespread species that is distributed from western Europe through the Caucasus and Siberia to the Far East (Sama 2002, Danilevsky 2018a). The species is ecologically associated with *Populustremula* but it can also develop in the wood of other tree species such as *Salix*, *Betula*, *Quercus*, *Malus*, *Sorbus*, *Robiniapseudoacacia*, *Fraxinus* and *Rosa* (Starzyk and Partyka 1993). According to Sama (2002), adults are active from April to August and can be found on their host plants or on the flowers of various plant species.

Only a single female was attracted to the artificial light source (Fig. 15H) at the edge of a mountain deciduous grove that consisted mainly of *Populus*, *Betula* and *Salix* (Fig. 15C).

#### Purpuricenini J. Thomson, 1861

##### Amarysius duplicatus

Taxon classificationAnimaliaColeopteraCerambycidae

Tsherepanov, 1980

Fig. 3H

###### Material examined.

East Kazakhstan Region: Putintsevo [Путинцево] env. (49°52'N, 84°21'E), 472 m a.s.l., 20 VI 2017, 1♀, leg. MW; 1♀, leg. MB, coll. LK.

###### Remarks.

*Amarysiusduplicatus* is a rather infrequent Siberian species that is distributed from West Siberia and eastern Kazakhstan to the Far East (Danilevsky 2018a). The species is ecologically associated with *Spiraea*, which is the only known host plant to date. The larvae feed and pupate in the wood of thin twigs. The adults are active from June to July and can be found on *Spiraea* flowers in large quantities (Cherepanov 1990b, Danilevskaya et al. 2009).

The first record for Kazakhstan was provided by Danilevskaya et al. (2009) based on several hundreds of specimens that were collected in the Putintsevo environs in June 2005. According to these authors, the species had been collected earlier near Ust-Kamenogorsk by I. Kostin in 1960 – misidentified as *Amarysiusaltajensis* (Laxmann, 1770) – and also by A. Napolov in 1994, but the record was not published.

Only two rather old females (one specimen lacked several segments of both antennae) were collected on the leaves of faded *Spirea* shrubs in a mixed forest that covers the foothills of the West Altai Mountains. Taking into account the condition of host plants and specimens, as well as the number of individuals that had been collected here in June 2005, it must have been the end of the appearance of this species.

##### Anoplistes galusoi

Taxon classificationAnimaliaColeopteraCerambycidae

(Kostin, 1974) *

Figs 3C, D , 10E, F

###### Material examined.

Almaty Region: 25 km SW of Kalinino [Басши], 691 m a.s.l., 13 VI 2017, 1♂, leg. LK; 2♂♂, 1♀, leg. WTS.

###### Remarks.

This is an endemic Kazakh species with its known distribution limited to the area of Mt. Ulkunkalkan at the Ili River in the southeastern part of the country (Kostin 1974, Danilevsky 2018a). According to Kostin (1974), the larvae develop in the roots and basal parts of the stems of living *Ephedrastrobilacea*. The irregular feeding ground is widened with the increasing of larva and although it is initially oriented downwards, it turns back to the top. Its length usually does not exceed 20 cm. Most of the emergence holes of the adults are located at the base of the root neck, approx. a few centimetres above ground level. The damaged shoots dry up before the autumn, and sometimes this may cause the whole bush to die. Adults that are rather immobile can be found from the end of May to mid-June. They copulate on host plants shortly after they hatch and supplementary feeding does not seem to be important in this species.

The imagines (Fig. 10E, F) were observed during windy and hot weather sitting on tufts of *E.strobilacea* (Fig. 10G) that were growing on the steep mountain slopes on the western side at a higher altitude in the habitat of a stony semi-desert that was sparsely covered with vegetation (Fig. 10H). Only four inactive specimens (three males and one female) were found despite checking nearly a thousand *Ephedra* shrubs. They did not seem to react to either the strong gusts of wind or the presence of observers. The beetles were found throughout most of the day from about 11 a.m. to 6 p.m. This strongly limited, endemic species seems to be in decline recently. In order to protect the exact locality of this vulnerable cerambycid, even approximate geographical coordinates have not been given. The species may somehow be related to the extremely rare *Anoplistesdiabolicus* Reitter, 1915.

##### Anoplistes halodendri halodendri

Taxon classificationAnimaliaColeopteraCerambycidae

(Pallas, 1773)

Fig. 3E–G

###### Material examined.

East Kazakhstan Region: 15 km W of Tarbagatay [Тарбагатай] (47°46'N, 81°37'E), 1072 m a.s.l., 15 VI 2017, 1♂, leg. LK; 20 km W of Tarbagatay [Тарбагатай] (47°47'N, 81°42'E), 1000 m a.s.l., 16 VI 2017, 1♀, leg. MW; Zhantikei [Жәнтікей] env. (48°04'N, 82°42'E), 455 m a.s.l., 16 VI 2017, 1♂, leg. MW; 10 km S of Bayash Utepov [Баяш Утепов] (49°35'N, 82°28'E), 508 m a.s.l., 25 VI 2017, 11♂♂, 5♀♀, leg. MW.

###### Remarks.

*Anoplisteshalodendri* is an east-Palaearctic species that is distributed from the Balkans to the Russian Far East, China, Korea and Japan (Danilevskaya et al. 2009). Within its range, it was divided into seven subspecies: *A.h.balcanicus* Sláma, 2010, *A.h.ephippium* (Steven & Dalman, 1817), *A.h.halodendri*, *A.h.heptapotamicus* (Semenov, 1926), *A.h.kasatkini* Lazarev, 2014, *A.h.minutus* Hammarström, 1892 and *A.h.pirus* (Arakawa, 1932). Apart from its nominotypical form, two other subspecies are known to occur in Kazakhstan: *A.h.ephippium* and *A.h.heptapotamicus* (Danilevsky 2018a). According to Cherepanov (1990b), the larvae of this species are ecologically associated with deciduous trees and shrubs (e.g. *Acacia*, *Daphnemezereum*, *Quercus* and *Salix*) in steppe and forest-steppe habitats. The adults are active from July.

In the Sibinka River valley, the species was collected numerously in 2002 and as a single specimen in 2005 under the same conditions (Danilevskaya et al. 2009). According to these authors, this population can be considered typical.

All of the individuals were collected on the pea shrub *Caragana* spp. (Fig. 11H). Only three rather fresh specimens were found in mid-June in the area of Tarbagatay (Fig. 11G) (each at a different locality and on two different species of *Caragana*) despite a long and attentive investigation of several suitable plots with numerous pea shrub bushes, which might suggest the beginning of the appearance of this species. On the other hand, nine days later in the Sibinka River valley (Fig. 15B), about a dozen very damaged specimens were observed gathered on a single pea shrub while no additional specimens were found on the neighbouring shrubs. Since the two pairs were observed *in copula*, it is probable that the males that still survived were attracted by the last females. This, in turn, clearly indicated the end of appearance; however, it may be related to the difference in the altitude of these localities (Tarbagatay approx. 1000 m and Sibinka 455 m a.s.l.). It is worth noting that neither during the presented expedition, nor in Mongolia (2015), were any imagines or immature stages of this species found on any different host or in any different habitat without *Caragana*, despite the thorough investigations of many plots and plant species using various methods. A similar observation concerning the occurrence of this species solely on *Caragana* spp. was made by Danilevsky (2018c) in Kazakhstan and Mongolia. It is possible that all of the records that are connected to other host plants may refer to related taxa, e.g. *A.h.ephippium*, or that some sibling species exist in this group, similar to the genus *Amarysius*. Furthermore, regarding the subspecies heptapotamicus, which was described from SE Kazakhstan (Lake Balkhash and Tarbagatay env.) based on several rather strange specimens, no significant morphological differences have been found between the specimens from the Sibinka River valley (Fig. 3E) and the Tarbagatay environs (Fig. 3F). However, some individuals from the area of Lake Balkhash should also be studied.

##### Anoplistes jacobsoni

Taxon classificationAnimaliaColeopteraCerambycidae

Baeckmann, 1904 *

Figs 3A, B , 11A–C

###### Material examined.

Kyzylorda Region: Tartogay env. [Тартогай] (44°25'N, 66°13'E), 135 m a.s.l., 7 VI 2017, 22♂♂, 11♀♀, leg. LK; 38♂♂, 16♀♀, leg. WTS; 20♂♂, 5♀♀, leg. MB; 40♂♂, 17♀♀, leg. MW.

###### Remarks.

This is an endemic Kazakh species that is only known from several localities along the lower and middle course of the Syr Darya River in the southern part of the country. According to Plavilstshikov (1940), *A.jacobsoni* is ecologically associated with *Tamarix* and *Elaeagnus*. However, Kostin (1974) and Kadyrbekov et al. (1996) barely mentioned the larval development in *Halimodendron*. Many aspects of the species biology including the duration of its life cycle remain unknown. The adults appear from the end of May to June and can be found on host plants (Kadyrbekov et al. 1996).

Our own observations clearly indicate that Plavilstshikov’s data (1940) regarding the host plants are wrong. The species was observed in large numbers in a tugay habitat with *Halimodendron*, *Tamarix* and *Elaeagnus* (Fig. 11D). *Anoplistesjacobsoni* is ecologically associated with the common salt tree *Halimodendronhalodendron* (Fig. 11E) and none of the individuals were observed on blossoming tamarisks or on oleasters despite the significant share of these plants in the habitat. Additionally, no emergence holes matching this longhorn beetle were found on the two last plant species mentioned, unlike the common salt tree on which many of them were observed. Therefore, Plavilstshikov probably recorded the main woody plants that formed the tugays in the habitat on which the species was found. According to our observations and based on the distribution of certain plant species, *A.jacobsoni* seems monophagous on *Halimodendronhalodendron*. The larvae and feeding galleries (Fig. 11F) of this species were found in the stems and branches 2 to 5 cm in diameter. The adult emergence holes were located at heights that ranged from approx. 10 cm to 1 m above ground level. Although they were not usually concentrated, sometimes a few of them were situated about a dozen cm from each other. The larvae initially feed on living shoots, which died afterwards. The imagines were observed during a scorching (35 °C) day from around 10:30 a.m., when the males (Fig. 11A) were already actively but rather slowly flying in the upper parts of the most impressive shrubs (Fig. 11E). Most of females (Fig. 11B) that were collected were sitting on the shady leaves of the lower branches. However, some copulating pairs (Fig. 11C) were spotted before noon as well. The highest activity of the beetles occurred around noon and although the flight of the adults began to end after that, some individuals were still found sitting on the leaves and branches. At around 1 p.m., the number of visible individuals quickly started to decrease until about 1:30 p.m. when most of the beetles were already hidden from the heat. No adults were observed again until late in the afternoon (about 4 p.m.) and as time passed, their numbers began to gradually increase. Most of the specimens were collected around 5–6 p.m. while they sitting or copulating in different parts of shrubs, mostly in exposed places but sometimes also in the shade. At that time, most of the mating couples were observed; however, despite the largest number of individuals, not many were actively flying. In the evening hours, the beetles started to hide again so the last specimens were observed before 8 p.m. This was clearly the climax of the appearance of this species despite the fact that the *Halimodendron* shrubs were already faded at that time. The males prevailed in the population (ratio of approx. 2:1). Nevertheless, freshly emerged individuals were also found. *Anoplistesjacobsoni* was observed sympatrically with *Chlorophoruselaeagni*, which visited the blossoming tamarisks exclusively.

#### Tillomorphini Lacordaire, 1868

##### Cleroclytus semirufus collaris

Taxon classificationAnimaliaColeopteraCerambycidae

Jakovlev, 1885

Fig. 3I–K

###### Material examined.

Almaty Region: Kabanbay [Қабaнбaй] (45°50'N, 80°37'E), 661 m a.s.l., 8 V 2017, 1♂ (at light), leg. GT, coll. RP; 7 km W of Kabanbay [Қабaнбaй] (45°48'N, 80°31'E), 720 m a.s.l., 9 V 2017, 3♂♂, 2♀♀, leg. RP; 12♂♂, 29♀♀, leg. JH; 2♂♂, 4♀♀, leg. KL; 13 km W of Szonży [Шонжы] (43°32'N, 79°17'E), 730 m a.s.l., 12 V 2017 (9–30 XI 2017, ex cult.) 2♂♂, 6♀♀, from *Berberisvulgaris*, leg. RP; (I 2018, ex cult.) 1♀, from *Fraxinussogdiana*, leg. JH; (XII 2017, ex cult.) 1♂, 1♀, from *Elaeagnusangustifolia*, leg. KL; Kapchagay [Қапшағай] (43°52'N, 77°03'E), 610 m a.s.l., 1 V 2017 (IX–X 2017, ex cult.) 1♂, 4♀♀, from *Acer* sp., leg. JH; East Kazakhstan Region: 20 km NW of Zaysan [Зайсан] (47°34'N, 84°39'E), 453 m a.s.l., 6 V 2017, 1♀, leg. JH; Aynabulak [Aынабyлaқ] ad Zaysan [Зайсан] (47°33'N, 85°03'E), 508 m a.s.l., 7 V 2017 (26 IX–17 X 2017, ex cult.) 13♂♂, 9♀♀, from *E.angustifolia*, leg. RP.

###### Remarks.

*Cleroclytussemirufus* is distributed from Central Asia and Afghanistan to Mongolia and northwestern China. Three subspecies have been described to date: *C.s.semirufus* Kraatz, 1884, *C.s.collaris* Jakovlev, 1885 and *C.s.savitsky* Lazarev, 2014. The subspecies discussed here is known to occur in Afghanistan, Kirgizia, Kazakhstan and the Xinjiang region in China (Danilevsky 2001a, 2018a). According to the literature, it inhabits various habitats such as mountain and submountain forests (Plavilstshikov 1940), steppes (Kadyrbekov and Tleppaeva 1997) and tugay forests (Borissova 2018). The species is strongly polyphagous; its larvae develop in the twigs, *inter alia*, of *Salix*, *Populus* and *Rosa* (Kadyrbekov and Tleppaeva 1997, Ishkov and Kadyrbekov 2004). The imagines are active from May to July and can be found on their host plants as well as on various blossoming plant species (Plavilstshikov 1940).

Numerous individuals were collected on the flowers of the hoary cress *Lepidiumdraba* (Fig. 12E). Additionally, many specimens were reared from the inhabited material of various plant species: *Berberisvulgaris* (Fig. 12F), *Fraxinussogdiana*, *Elaeagnusangustifolia* and *Acer* sp. The wood collected in Aynabulak turned out to be sympatrically infested with the larvae of two rather rare Bostrichidae species (det. et coll. J. Borowski): *Enneadesmusscopini* (Fursov, 1936) (> 30 exx.) and *Lyctusturkestanicus* (Lesne, 1935) (2 exx.).

### Lamiinae Latreille, 1825

#### Acanthoderini J. Thomson, 1860

##### Aegomorphus clavipes

Taxon classificationAnimaliaColeopteraCerambycidae

(Schrank, 1781)

###### Material examined.

East Kazakhstan Region: Putintsevo [Путинцево] env. (49°52'N, 84°21'E), 472 m a.s.l., 19–23 VI 2017, 18♂♂, 7♀♀, leg. WTS; 4♂♂, 2♀♀, leg. LK; 16♂♂, 20♀♀, leg. MW; 3♂♂, 2♀♀, leg. MB; Bykovo [Быково] env. (49°39'N, 84°33'E), 570 m a.s.l., 24 VI 2017, 2♂♂, 1♀, leg. WTS; 5♂♂, 7♀♀, leg. LK; 3♂♂, 2♀♀, leg. MW.

##### Aegomorphus obscurior

Taxon classificationAnimaliaColeopteraCerambycidae

(Pic, 1904)

Fig. 4D

###### Material examined.

East Kazakhstan Region: Putintsevo [Путинцево] env. (49°52'N, 84°21'E), 472 m a.s.l., 21–24 VI 2017, 1♂, leg. WTS; 3♀♀, leg. LK; 1♂, leg. MW.

###### Remarks.

This species is currently known to be broadly distributed in Russia and in the Siberian part of Kazakhstan (Danilevsky and Shapovalov 2007) as well as in Mongolia (Hilszczański 2008). In Europe, it reaches Latvia (Telnov 2016) and eastern Poland (Hilszczański 2008). *A.obscurior* was discussed in a previous paper concerning the longhorn beetles of Mongolia (Karpiński et al. 2018).

Several specimens were beaten down from the branches and thin shoots of birches on an exposed site next to a river in a mountain deciduous forest dominated by *Populus* and *Salix* with an admixture of *Betula* (Fig. 15D). In this region, the species is ecologically associated with birch, in contrast to its western boundary of occurrence (e.g. Poland), where all records are related to oak. We observed this species together with *A.clavipes*, which was definitely more numerous and was mainly found on poplars and willows.

#### Agapanthiini Mulsant, 1839

##### Agapanthia alternans alternans

Taxon classificationAnimaliaColeopteraCerambycidae

Fischer von Waldheim, 1842

Fig. 5D, E

###### Material examined.

East Kazakhstan Region: 5 km SE of Kuygan [Құйған] (48°38'N 83°32'E), 439 m a.s.l., 18 VI 2017, 1♂, 1♀, leg. MB; 1♂, leg. WTS; 7 km N of Samarskoje [Самарское] (49°05'N, 83°20'E), 626 m a.s.l., 18 VI 2017, 3♂♂, 1♀, leg. WTS; 1♂, leg. LK; Putintsevo [Путинцево] env. (49°52'N, 84°21'E), 472 m a.s.l., 22 VI 2017, 1♂, leg. WTS; 1♂, leg. MW; Bykovo [Быково] env. (49°39'N, 84°33'E), 570 m a.s.l., 24 VI 2017, 2♂♂, leg. WTS; 1♂, 4♀♀, leg. LK; 2♂♂, 2♀♀, leg. MW.

###### Remarks.

This is a rather widespread species that is distributed from the central part of Kazakhstan to East Siberia. Five subspecies have been described, four of which are known to occur in Kazakhstan: *A.a.alternans* Fischer von Waldheim, 1842, *A.a.paralternans* (Danilevsky, 2017), *A.a.songarica* Kostin, 1973 and *A.a.tarbagataica* Kostin, 1978 (Danilevsky 2018a). Its nominotypical form occupies the northern part of the species range from about NE Kazakhstan to Transbaikalia and Mongolia. The species is ecologically associated with *Prangos* and *Ferula* (Danilevsky 2017).

Several specimens of this taxon were collected in the Putintsevo environs in June 2005 by Danilevskaya et al. (2009), however the subspecies was not specified.

The specimens were collected in rather different habitats, such as the roadside strip with herbaceous vegetation at the edge of a coniferous forest (Fig. 12H), in the entire area of Putintsevo and Bykovo. It was observed sympatrically with *Agapanthiadahlicalculensis*, similar to Danilevskaya et al. (2009); however, according to these authors, both of these species have different host plants.

##### Agapanthia dahli calculensis

Taxon classificationAnimaliaColeopteraCerambycidae

Lazarev, 2013 *

Fig. 5A–C

###### Material examined.

Almaty Region: 5 km N of Karashota [Каражота] (43°41'N, 78°09'E), 492 m a.s.l., 3 VI 2017, 1♀, leg. WTS; East Kazakhstan Region: Putintsevo [Путинцево] env. (49°52'N, 84°21'E), 472 m a.s.l., 22 VI 2017, 1♂, leg. WTS; 1♂, leg. MW; Bykovo [Быково] env. (49°39'N, 84°33'E), 570 m a.s.l., 24 VI 2017, 3♂♂, leg. WTS; 1♀, leg. LK; 6♂♂, 5♀♀, leg. MW; 10 km S of Bayash Utepov [Баяш Утепов] (49°35'N, 82°28'E), 508 m a.s.l., 25 VI 2017, 2♂♂, 1♀, leg. MW.

###### Remarks.

This is an extremely polymorphic and widespread species that is distributed from Central and Southern Europe through Asia Minor, the Caucasus and Central Asia to almost entire region of Siberia (Danilevsky 2018a). Within its range, it is represented by many distinct local forms. To date, as many as 15 subspecies have been designated. Most of them were described or moved from the species level very recently (Lazarev 2013a,c, Lazarev et al. 2016). *Agapanthiadahlicalculensis* is endemic to northeastern Kazakhstan (Lazarev 2013a). According to this author, the subspecies primarily differs in the poorly developed setae tufts of its antennal joints, which are very long and dense in all of the other geographical forms as well as in the poorly pubescent elytra. Although *Agapanthiadahli* is ecologically associated with various herbaceous plants species, the larvae prefer to feed in the inner tissues of stems of *Cirsium*, *Melilotus*, *Cannabis* and *Ferula*. On the other hand, the series of type specimens of *A.d.calculensis* were collected on *Malva* sp. and *Dictamnus* sp. (Lazarev 2013a). The life cycle of this species usually lasts one year but sometimes can be extended to two years (Cherepanov 1991a).

Several specimens of this taxon were collected in the Putintsevo and the Sibinka River valley environs in June 2005 before *A.d.calculensis* was described; hence, it was recorded as a nominotypical subspecies by Danilevskaya et al. (2009).

According to M. Danilevsky (2018, pers. comm.), the single female (Fig. 5C) that was collected on *Carduus* sp. in the area of Karashota may represent a new subspecies. However, more specimens, including males, need to be gathered to support this hypothesis.

##### Agapanthia villosoviridescens

Taxon classificationAnimaliaColeopteraCerambycidae

DeGeer, 1775 

###### Material examined.

East Kazakhstan Region: Bykovo [Быково] env. (49°39'N, 84°33'E), 570 m a.s.l., 24 VI 2017, 1♀, leg. LK; 1♂, leg. MW.

###### Remarks.

This is a typical Palaearctic species that is distributed from Southern Europe to East Siberia and Mongolia (Danilevsky 2018a). The larvae feed in the inner tissues of the stems of various herbaceous plants, mainly on *Carduus*, *Cirsium* and *Urtica*, and less frequently on *Angelica*, *Chaerophyllum*, *Eupatorium*, *Heracleum*, *Senecio*, *Scrophularia* and *Anthriscus* (Cherepanov 1991a). The adults can be found on their host plants from May to August.

One of the collected specimens represented a very rare and interesting form with red coloured antennae. Such forms are also known to occur in Europe.

##### Agapanthia violacea

Taxon classificationAnimaliaColeopteraCerambycidae

Fabricius, 1775

###### Material examined.

Almaty Region: 7 km W of Kabanbay [Қабaнбaй] (45°48'N, 80°31'E), 720 m a.s.l., 9 V 2017, 9♂♂, 4♀♀, leg. RP & TJ; 9♂♂, 6♀♀, leg. JH; 3♀♀, leg. KL; 1♂, leg. GT; 6 km E of Koylik [Қoйлық] (45°38'N, 80°19'E), 737 m a.s.l., 9 V 2017, 1♂, leg. RP; 2♂♂, leg. KL; East Kazakhstan Region: Bykovo [Быково] env. (49°39'N, 84°33'E), 570 m a.s.l., 24 VI 2017, 3♂♂, leg. WTS.

###### Remarks.

*Agapanthiaviolacea* is distributed from Southern and Central Europe through Asia Minor and the Caucasus to Lake Baikal in Siberia. It is a thermophilic species that mainly inhabits xerothermic sites. It is ecologically associated with various plant species, mainly of Apiaceae and Asteraceae. The adults can be found on their host plants from mid-May to July (Paulus 1974, Cherepanov 1991a).

The specimens were mainly collected on *Astragalussieversianus* (Fig. 12G).

##### Agapanthiola leucaspis

Taxon classificationAnimaliaColeopteraCerambycidae

(Steven, 1817)

###### Material examined.

Almaty Region: 2 km E of Saryozek [Сарыөзек], (44°22'N, 78°01'E), 875 m a.s.l., 2 VI 2017, 1♂, 1♀, leg. WTS; 1♀, leg. MW; East Kazakhstan Region: 15 km NW of Taskesken [Таскескен] (47°18'N, 80°36'E), 15 VI 2017, 627 m a.s.l., 3♂♂, 2♀♀, leg. WTS; 1♂, 1♀, leg. LK; 1♂, leg. MW; Bykovo [Быково] env. (49°39'N, 84°33'E), 571 m a.s.l., 21 VI 2017, 1♀, leg. WTS.

###### Remarks.

*Agapanthiolaleucaspis* is a west-Palaearctic species that is distributed from Southern Europe through the southern regions of Eastern Europe and Russia, Turkey, Central Asia to Lake Baikal in Siberia, Mongolia and China (Sama 2002, Danilevsky 2018a). It is a polyphagous species whose larvae develop in various herbaceous plants, e.g. *Melilotusofficinalis*, *Campanulasibirica*, *Salviastepposa* and *Erigeron* sp. (Cherepanov 1991a).

Several specimens were collected in rather different habitats (e.g. roadside vegetation strip, mountain forest) using the sweep-netting method.

#### Desmiphorini J. Thomson, 1860

##### Rhopaloscelis unifasciatus

Taxon classificationAnimaliaColeopteraCerambycidae

Blessig, 1873

Fig. 4E, F

###### Material examined.

East Kazakhstan Region: Putintsevo [Путинцево] env. (49°52'N, 84°21'E), 472 m a.s.l., 22–23 VI 2017, 2♀♀, leg. WTS; 1♂, leg. MB, coll. LK; 1♂, 1♀, leg. MW.

###### Remarks.

This is an east-Palaearctic species that is distributed from Altai to Sakhalin and Japanese islands, including China and the Korean peninsula (Cherepanov 1991a, Danilevsky 2018a). It is a highly polyphagous species whose larvae develop in the twigs or thin shoots of various deciduous trees and shrubs; however, they prefer *Salix*, *Morus*, *Acer*, *Aralia* and *Ulmus*. The larvae feed very intensely in the upper layers of wood and create longitudinal, often densely arranged, larval feeding galleries that are filled with fine sawdust. The life cycle lasts about two years. The imagines are active from May to July. The adults conduct their supplementary feeding on the young and thin twigs or branches of various deciduous species (Cherepanov 1991a).

*Rhopaloscelisunifasciatus* was recently reported from Kazakhstan for the first time by Danilevskaya et al. (2009) based on a single specimen that had accidently been collected on a stem of *Artemisia* in the Putintsevo environs.

In our research, several specimens were beaten down from the dead parts of young willows in a mountain deciduous forest dominated by *Populus* and *Salix*. These findings confirm the presence of this species in Kazakhstan. *Rhopaloscelisunifasciatus* shares the same habitat with other Lamiinae species, *inter alia*, *Exocentrusstierlini* and *Saperdasimilis* as well as with other saproxylic beetles, e.g. *Kolibaciasquamulata* (Gebler, 1830) (Trogossitidae) (Szczepański et al. 2018).

#### Dorcadionini Swainson, 1840

##### Dorcadion abakumovi sarkandicum

Taxon classificationAnimaliaColeopteraCerambycidae

Danilevsky, 2004 *

Fig. 6A, B

###### Material examined.

Almaty Region: 10 km SW of Sarkan [Cарқaн] (45°21'N, 79°48'E), 990 m a.s.l., 4 V 2017, 2♂♂, leg. RP; 1♂, 1♀, leg. KL; 1♂, leg. GT.

###### Remarks.

This taxon is endemic to eastern Kazakhstan and is known to occur only in one locality near the city of Sarkan in the foothills of the Dzungarian Alatau (Toropov and Milko 2013). This species includes four subspecies: *D.a.abakumovi* Thomson, 1865, *D.a.laterale* Jakovlev, 1895, *D.a.lepsyense* Danilevsky, 2004 and *D.a.sarkandicum*, which are represented only by small populations that occur in a limited area in the northern part of the Dzungarian Alatau in eastern Kazakhstan (Danilevsky 2004). The biology of the species is poorly known. The beetles occur at altitudes of approx. 800–1300 m a.s.l. where they feed on various species of Poaceae (Toropov and Milko 2013).

##### Dorcadion absinthium ishkovi

Taxon classificationAnimaliaColeopteraCerambycidae

Kadyrbekov, 2004 *

Fig. 7A, B

###### Material examined.

Almaty Region: 2 km E of Arkhaly [Apқaлы] (44°10'N, 77°56'E), 1005 m a.s.l., 2 V 2017, 6♂♂, 2♀♀, leg. RP; 11♂♂, 3♀♀, leg. KL.

###### Remarks.

*Dorcadionabsinthiumishkovi* is an endemic Kazakh taxon with its known distribution limited to an area situated approx. 50 km north of Kapchagay in the environs of Kerbulak (Kadyrbekov 2004, Toropov and Milko 2013). The biology of this species is poorly known. According to Kadyrbekov (2004), it inhabits the sandy desert above a canyon of the Ili River. Based on the known collection data, the imagines are active at the turn of April and May.

The population, which was dominated by males, was found in a steppe habitat (Fig. 13A) in the environs of Arkhaly. Our finding extends the known range of this taxon about 50 km to the east.

##### Dorcadion acutispinum

Taxon classificationAnimaliaColeopteraCerambycidae

Motschulsky, 1860 *

Figs 6G, H , 13B

###### Material examined.

Almaty Region: 16 km NE of Kapal [Қапал] (45°12'N, 79°14'E), 1275 m a.s.l., 3 V 2017, 14♂♂, 5♀♀, leg. RP; 6♂♂, 2♀♀, leg. JH; 3♂♂, leg. GT; 22 km E of Kapal [Қапал] (45°13'N, 79°16'E), 1201 m a.s.l., 3 V 2017, 4♂♂, 1♀, leg. KL; 15 km E of Kapal [Қапал] (45°11'N, 79°12'E), 1320 m a.s.l., 3 V 2017, 2♂♂, leg. KL; 34 km W of Kapal [Қапал] (45°14'N, 78°39'E), 665 m a.s.l., 3 V 2017, 1♂, leg. KL.

###### Remarks.

*Dorcadionacutispinum* is endemic to eastern Kazakhstan where it is known to occur in a few localities in the valley of the Kapal River and its surroundings in the northern range of Dzungarian Alatau (Danilevsky 1996c). The species inhabits sparse grasslands with sandy plots in a river valley. The imagines are active at the turn of April and May (Toropov and Milko 2013).

Numerous individuals were collected in steppe-like habitat in the Kapal canyon. The population was dominated by males (Fig. 13B) (ratio of approx. 3:1).

##### Dorcadion arietinum arietinum

Taxon classificationAnimaliaColeopteraCerambycidae

Jakovlev, 1898 *

Figs 6L , 13C

###### Material examined.

Almaty Region: 2 km N of Kegen [Қеғен] (43°02'N, 79°13'E), 1809 m a.s.l., 12 VI 2017, 7♂♂, leg. WTS; 3♂♂, leg. LK; 4♂♂, leg. MW.

###### Remarks.

*Dorcadionarietinum* includes seven described subspecies that are distributed in southern and southeastern Kazakhstan and northwestern China. The nominotypical subspecies is known to occur only in the area of SE Kazakhstan (Danilevsky 2018a). It inhabits mountain valleys and semi-arid areas at altitudes of approx. 600–2000 m a.s.l. The larvae feed on the roots of various grass Poaceae (e.g. *Stipa* spp.) and sedges of the Cyperaceae species. The adults feed on the aboveground parts of their host plants (Toropov and Milko 2013).

The males (Fig. 13C) were collected in a mountain steppe habitat sympatrically with *Dorcadioncrassipescrassipes* Ballion, 1878 and *Dorcadionsemenovisemenovi* Ganglbauer, 1884. Although the plot (Fig. 13D) was mainly covered with high tufts of grass, there were also large, bare sandy spots where reddish and rather active beetles were clearly visible.

##### Dorcadion arietinum charynense

Taxon classificationAnimaliaColeopteraCerambycidae

Danilevsky, 1996 *

###### Material examined.

38 km SW of Szonży [Шонжы] (43°21'N, 79°03'E), 1077 m a.s.l., 11 V 2017, 1♂, leg. GT.

###### Remarks.

The taxon is endemic to southeastern Kazakhstan. The only known population is distributed along the northern foot of the east part of the Turaigyr Mountains (Danilevsky 1996d). The biology is similar to the nominotypical subspecies.

Only a single, rather old male specimen was found in the Sharyn Canyon.

##### Dorcadion crassipes crassipes

Taxon classificationAnimaliaColeopteraCerambycidae

Ballion, 1878 *

Fig. 6E, F

###### Material examined.

Almaty Region: 40 km SE of Sary-Ozek [Сары-Озек] (44°13'N, 78°30'E), 1534 m a.s.l., 10 V 2017, 1♂, leg. GT, coll. RP; 2 km N of Kegen [Қеғен] (43°02'N, 79°13'E), 1809 m a.s.l., 12 VI 2017, 2♂♂, leg. MB, coll. LK & WTS.

###### Remarks.

*Dorcadioncrassipes* is distributed in southeastern Kazakhstan, northwestern China and Kyrgyzstan. Three subspecies have been described to date: *D.c.crassipes*, *D.c.glazunovi* Suvorov, 1910 and *D.c.validipes* Jakovlev, 1906. The nominotypical form is endemic to SE Kazakhstan and is known to occur eastwards from about the Chu-Ili Mountains to the Dzungarian Alatau (Danilevsky 1996b, 2018a). This is the most variable species of the *Compsodorcadion* group, which also includes *Dorcadionganglbaueri* Jakovlev, 1898, *Dorcadioncephalotes* Jakovlev, 1889 and *Dorcadiongebleri* Kraatz, 1873. According to Toropov and Milko (2013), both adults and larvae are ecologically associated with the needle grass of the genus *Achnatherum*, especially with *Achnatherumsplendens* (= *Stipasplendens* = *Lasiagrostissplendens*). Additionally, Danilevsky (1996b) claims that all of the taxa of the former *Compsodorcadion* subgenus are related to the grasses of the genus *Lasiagrostis*.

Despite several hours of searching at the locality near Kegen, only two males were collected in a mountain steppe habitat sympatrically with *Dorcadionarietinumarietinum* and *Dorcadionsemenovisemenovi* Ganglbauer, 1884. This seems to confirm the interesting observations of Danilevsky (1996b) that although two or three *Dorcadion* species often occur together in the same locality, they are never two species of the same subgenus. Similarly, in our study three species represented three different subgenera: *Acutodorcadion* Danilevsky, Kasatkin & Rubenyan, 2005, *Cribridorcadion* Pic, 1901 and *Dorcadion* s. str. Dalman, 1817. This new locality in the environs of Kegen is the southeasternmost known location of this species, which is situated more than 100 km from the nearest sites that are already known. The plot (Fig. 13D) was mainly covered with high tufts of grass.

##### Dorcadion gebleri gebleri

Taxon classificationAnimaliaColeopteraCerambycidae

Kraatz, 1873

Figs 6I–K , 13E

###### Material examined.

East Kazakhstan Region: 5 km SE of Kabanbay [Қабaнбaй] (47°49'N, 83°37'E), 461 m a.s.l., 6 V 2017, 2♂♂, leg. RP; 2♂♂, leg. JH; 1♂, 3♀♀, leg. KL; 1♀, leg. GT; 20 km NW of Zaysan [Зайсан] (47°34'N, 84°39'E), 453 m a.s.l., 6 V 2017, 2♂♂, leg. RP; 1♀, leg. JH; 1♀, leg. KL; 5 km NE of Zaysan [Зайсан] (47°30'N, 84°57'E), 509 m a.s.l., 17 VI 2017, 1♂ (dead specimen), leg. WTS; 1♂ (body remains), leg. MB, coll. LK; 1♂, 1♀ (body remains), leg. MW.

###### Remarks.

*Dorcadiongebleri* is distributed in eastern Kazakhstan and northwestern China. The species includes four subspecies: *A.g.demimetrum* Danilevsky, 1996, *A.g.gebleri*, *A.g.lukhtanovi* Danilevsky, 1996 and *A.g.takyr* Danilevsky, 1996. Most of them are endemic to E Kazakhstan; only the nominotypical form extends its range into China (Danilevsky 2018a). This taxon mainly occurs on a small area around Lake Zaysan. The imagines are active rather early from the end of April to May, and sometimes even in June (Danilevsky 1996a). According to Danilevsky (1996b), this is the largest representative of the entire *Dorcadion* genus. The larvae feed on the roots of *Achnatherium* spp., especially on *A.splendens*. The adults feed on the above-ground parts of their host plants on which they also copulate (Toropov and Milko 2013).

Several males and females (Fig. 13E) that were collected in May were observed in pasture habitats overgrown by high tufts of *Festuca* sp. (Fig. 13F). The specimens that were found in mid-June consisted of rather old body remains, which confirms the very early period of the occurrence of this species. The plot was also overgrown by high tufts of *Festuca* sp. The two males that were depicted (Fig. 6I, J) were collected in two different plots located approx. 100 km from each other.

##### Dorcadion gebleri lukhtanovi

Taxon classificationAnimaliaColeopteraCerambycidae

Danilevsky, 1996 *

###### Material examined.

East Kazakhstan Region: Kurshim [Күршім] env. (48°34'N, 83°36'E), 406 m a.s.l., 17 VI 2017, 1♂ (body remains), leg. WTS.

###### Remarks.

*Dorcadiongeblerilukhtanovi* is known from several localities eastwards from Lake Zaysan in northeastern Kazakhstan (Toropov and Milko 2013). It occurs in the Kurchum Mountain ridge and in the surrounding foothill area (Danilevsky 1996a). The larvae probably feed on the roots of *Achnatherium* spp. The imagines are active from the end of April to May and only sometimes can be observed in June (Toropov and Milko 2013). According to Danilevsky (1996a), this taxon seems to be a transitional form between *D.g.gebleri* and *Dorcadioncephalotes* Jakovlev, 1890.

Only the remains of a single male were found in a grassy, semi-ruderal habitat in the Kurchum River valley.

##### Dorcadion kapchagaicum

Taxon classificationAnimaliaColeopteraCerambycidae

Danilevsky, 1996 *

Fig. 7C, D

###### Material examined.

Almaty Region: 8 km N of Kapchagay [Қапшағай] (43°56'N, 77°02'E), 610 m a.s.l., 1 V 2017, 7♂♂, 7♀♀, leg. RP; 9♂♂, 6♀♀, leg. JH; 10♂♂, 6♀♀, leg. KL; 1♂, leg. GT; 50 km N of Kapchagay [Қапшағай] (44°18'N, 76°56'E), 587 m a.s.l., 1 V 2017, 4♂♂, 3♀♀, leg. RP; 4♂♂, 2♀♀, leg. JH; 2♂♂, 6♀♀, leg. KL; 3♂♂, leg. GT.

###### Remarks.

*Dorcadionkapchagaicum* is endemic to southeastern Kazakhstan. The species is distributed in the area located to the north and west of the Kapchagay Reservoir, where it prefers clayey, semi-desert habitats in plains and foothills. The larvae feed on roots of various species of Poaceae. The beetles occur at altitudes of approx. 700 m a.s.l. where they feed on their host plants. The adults are active from the end of April to the beginning of June (Toropov and Milko 2013). According to Danilevsky (1996b), the most related taxon is *Dorcadiontschitscherini* Jakovlev, 1900, which is distributed slightly more to the south between the cities of Kapchagay and Almaty.

Numerous individuals were collected in steppe habitats in the area north of Kapchagay. At that time, the gender ratio of the observed population was rather equal.

##### Dorcadion morozovi

Taxon classificationAnimaliaColeopteraCerambycidae

Danilevsky, 1992

Fig. 8B

###### Material examined.

Almaty Region: 10 km N of Kegen [Қеғен] (43°09'N, 79°12'E), 1840 m a.s.l., 12 V 2017, 1♀, leg. JH, det. M. Danilevsky.

###### Remarks.

The species is endemic to southeastern Kazakhstan and Xinjiang province in China (Danilevsky 2018a). In Kazakhstan, it occurs locally in the environs of Kegen and Narynkol (Danilevsky 1992, Toropov and Milko 2013), where it inhabits valleys with steppe and meadow vegetation up to 2500 m a.s.l. The larvae feed on roots of various grass species (e.g. *Festucaspp*.). The imagines are active from mid-April to the end of May (Toropov and Milko 2013).

##### Dorcadion mystacinum rufidens

Taxon classificationAnimaliaColeopteraCerambycidae

Jakovlev, 1906 * 

###### Material examined.

Zhambyl Region: 10 km NW of Akkol [Акколь] (43°27N, 70°35'E), 382 m a.s.l., 5 VI 2017, 1♀ (body remains), leg. MB, coll. LK.

###### Remarks.

The species is distributed in south Kazakhstan and northwestern Kyrgyzstan (Toropov and Milko 2013). Three subspecies have been described to date: *D.mystacinummystacinum* Ballion, 1878, *D.mystacinumrufidens* and *D.mystacinumpumilio* Plavilstshikov, 1951 (Danilevsky 2018a). This subspecies seems to be endemic to the southeastern slopes of the Syr-Dar Karatau Mountains, where it mainly inhabits sparse grasslands and fixed sands in clayey and stony piedmonts between 320 and 1150 m a.s.l. The larvae feed on the roots of various grass species, mainly on *Festuca* and *Stipa*. The imagines are active from the end of April to the end of May (Toropov and Milko 2013).

Only the remains of a single female were found in a habitat with *Caragana* shrubs near the shore of a salt lake.

##### Dorcadion nikolaevi

Taxon classificationAnimaliaColeopteraCerambycidae

Danilevsky, 2005 *

Fig. 7E, F

###### Material examined.

Almaty Region: 6 km E of Koylik [Қoйлық] (45°38'N, 80°19'E), 737 m a.s.l., 4 V 2017, 15♂♂, 3♀♀, leg. RP; 6♂♂, 1♀, leg. JH; 1♂, 1♀, leg. GT; 9 V 2017, 9♂♂, leg. RP; 9♂♂, leg. JH; 7♂♂, 1♀, leg. KL.

###### Remarks.

The species is endemic to eastern Kazakhstan. It occurs only in the central part of the northern slopes of Dzungarian Alatau where it inhabits piedmont areas between 600–700 m a.s.l. The larvae feed on the roots of various grass species. The imagines are active mostly from the end of April to the second half of May (Toropov and Milko 2013).

##### Dorcadion semenovi semenovi

Taxon classificationAnimaliaColeopteraCerambycidae

Ganglbauer, 1884

Figs 8C, D , 13G

###### Material examined.

Almaty Region: 10 km N of Kegen [Қеғен] (43°07'N, 79°11'E), 1922 m a.s.l., 12 V 2017, 5♂♂, 8♀♀, leg. RP; 2♂♂, 2♀♀, leg. JH; 2♂♂, leg. KL; 17 km SE of Kegen [Қеғен] (42°55'N, 79°25'E), 2078 m a.s.l., 12 V 2017, 5♂♂; leg. KL; 15 km N of Kegen [Қеғен] (43°09'N, 79°12'E), 1844 m a.s.l., 11 V 2017, 3♀♀, leg. KL; 2♂♂, leg. GT; 5 km E of Saryzhaz [Сарыжаз] (42°55'N, 79°40'E), 1900 m a.s.l., 12 VI 2017, 2♂♂, 9♀♀, leg. WTS; 9♂♂, 6♀♀ (1♂, 4♀♀ – body remains), leg. LK; 5♂♂, 8♀♀, leg. MW; 2 km N of Kegen [Қеғен] (43°02'N, 79°13'E), 1809 m a.s.l., 12 VI 2017, 7♂♂, leg. WTS; 3♂♂, 1♀, leg. LK; 2♂♂, 2♀♀, leg. MW.

###### Remarks.

*Dorcadionsemenovi* is a very variable species, which includes ten subspecies that are distributed in the area of northern Kyrgyzstan, southeastern Kazakhstan and western China (Danilevsky 2002, 2018a). Its nominotypical subspecies is distributed to the northeast of Lake Issyk-Kul, mainly in the environs of the villages of Kegen and Narynkol (Danilevsky 2002, Toropov and Milko 2013). It inhabits foothills and river valleys at altitudes of 1600–3200 m a.s.l. The larvae feed on the roots of various grass species. The imagines are active from the end of April to the end of June, depending on the altitude on which they occur (Toropov and Milko 2013).

Most of the specimens were collected from a few different localities in the environs of Kegen. At a plot located 2 km N of Kegen, this species was observed in a mountain steppe habitat sympatrically with *D.crassipescrassipes* and *D.arietinumarietinum*. Although the plot (Fig. 13D) was mainly covered with high tufts of grass, there were also large, bare sandy spots. This species seems to have a long period of its occurrence. In May of the same year, numerous specimens were found in very good condition; however, over a month later, some live (although damaged) males (Fig. 13G) and females were still found.

##### Dorcadion sokolowi

Taxon classificationAnimaliaColeopteraCerambycidae

Jakovlev, 1899

Fig. 8E, F

###### Material examined.

Almaty Region: 7 km N of Sarymbel [Сарымбель] (44°29'N, 80°04'E), 1725 m a.s.l., 11 V 2017, 13♂♂, 6♀♀, leg. RP; 2♂♂, 2♀♀, leg. JH; 4♂♂, 4♀♀, leg. KL.

###### Remarks.

*Dorcadionsokolowi* is distributed in southeastern Kazakhstan and the Xinjiang province in China (Danilevsky 2018a). In Kazakhstan, it mainly occurs in the southeastern piedmonts of Dzungarian Alatau in environs of the town of Zharkent. It is also known from one locality in the valleys of the Ili and Charyn Rivers. The species inhabits stony foothills with a low-herb grassy vegetation at altitudes of approx. 600–700 m a.s.l. The larvae feed on the roots of various grass species. The adults are active from the end of April to the second half of May (Toropov and Milko 2013).

A rather unusual observation regarding the genus *Dorcadion* of a few specimens of both genders that were gathering on a single female probably in an attempt to copulate (Fig. 13H) was made in the area of Sarymbel.

##### Dorcadion songaricum

Taxon classificationAnimaliaColeopteraCerambycidae

Ganglbauer, 1884

Fig. 8A

###### Material examined.

East Kazakhstan Region: 10 km E of Kyzyl Kesik [Қызыл Қесиқ] (47°53'N, 82°06'E), 808 m a.s.l., 8 V 2017, 1♀, leg. RP, det. M. Lazarev.

###### Remarks.

*Dorcadionsongaricum* is distributed in east Kazakhstan and the Xinjiang province in China (Danilevsky 2018a). In Kazakhstan, the species is known from the northern slopes of the Tarbagatay and Saur Mountain ranges as well as the southern slopes of the Manrak Mountains. It inhabits sparse grasslands in piedmont regions. The larvae feed on the roots of various grass species. This is one of the latest occurring species; the adults are active from the second half of May to the end of June (Toropov and Milko 2013).

Only a single female was collected in a mountain-steppe habitat in the area of rocky hills (Fig. 14F).

##### Dorcadion suvorovi konyrolenum

Taxon classificationAnimaliaColeopteraCerambycidae

Danilevsky, 1996 *

Figs 7G, H , 14A

###### Material examined.

Almaty Region: Karlygash [Карлыгаш] env. (44°16'N, 78°28'E), 1398 m a.s.l., 2 VI 2017, 1♂, leg. WTS; 1♀, leg. MB, coll. LK.

###### Remarks.

The species includes five subspecies, which are mainly distributed within the area from the Kapchagay Reservoir to the Dzungarian Alatau (Toropov and Milko 2013). *Dorcadionsuvorovikonyrolenum* is an endemic taxon whose known localities are limited to the environs of Konyrolen in southeastern Kazakhstan (Danilevsky 1996c, Toropov and Milko 2013). The biology of the species is poorly understood. According to Toropov and Milko (2013), the larvae feed on the roots of various grass species. The beetles occur at altitudes between 600–1,800 m a.s.l. The adults are active from the end of April to the beginning of June.

Only a single male (Fig. 14A) and female were collected in a mountain steppe habitat. The plot (Fig. 14B) was mainly covered with medium-high grass. Such a small number of observed individuals may indicate the end of the appearance of this species.

##### Dorcadion tenuelineatum

Taxon classificationAnimaliaColeopteraCerambycidae

Jakovlev, 1895 *

Fig. 6C, D

###### Material examined.

Almaty Region: 10 km E of Gerasimovka [Герасимовка] (45°48'N, 80°59'E), 844 m a.s.l., 4 V 2017, 1♂, leg. RP; 1♂, leg. JH; 1♂, leg. KL; 9 V 2017, 12♂♂, 1♀, leg. RP; 3♂♂, 1♀, leg. KL.

###### Remarks.

*Dorcadiontenuelineatum* is a species that is endemic to Kazakhstan (Danilevsky 2018a), where it occurs only in the area between Lake Alakol and the northeastern range of the Dzungarian Alatau (Toropov and Milko 2013). The species inhabits grasslands on the slopes in piedmont areas at an altitude of approx. 1000 m a.s.l. The larvae feed on the roots of various species of Poaceae. The adults are active from the end of April to the second half of May (Toropov and Milko 2013).

##### Dorcadion tianshanskii radkevitshi

Taxon classificationAnimaliaColeopteraCerambycidae

Suvorov, 1910 *

Figs 7I, J , 14C

###### Material examined.

Zhambyl Region: 5 km W of Kenen [Кенен] (43°25'N, 74°58'E), 928 m a.s.l., 4 VI 2017, 9♂♂, 3♀♀ (5♂♂ – body remains), leg. LK; 3♂♂, 2♀♀, leg. WTS; 18♂♂, 11♀♀ (3♂♂, 2♀♀ – body remains), leg. MW; 1♂ (dead specimen), 1♀, leg. MB.

###### Remarks.

*Dorcadiontianshanskii* is a species that is endemic to Kazakhstan (Danilevsky 2018a). It includes eight subspecies, which are distributed in the area to the north of the Chu River, mainly in the environs of Kenen (Danilevsky 2012, Toropov and Milko 2013). The biology of this taxon is poorly known. According to Toropov and Milko (2013), it inhabits various habitats depending on the subspecies. The larvae feed on the roots of various grass species. The imagines are active from the end of April to the end of May.

The individuals of *D.t.radkevitshi* were collected during relatively cold (approx. 20 °C) and cloudy weather in a grassland habitat (Fig. 14D). Despite the rather late period for the species, a few copulating pairs as well as some males, which were actively moving between tufts of grass, were still found. The females (Fig. 14C) were found in rather better condition. Nevertheless, many specimens were already dead. The individuals that were still alive occurred more frequently in a shallow depression near a small stream, usually no further than 1.5 m from the stream.

##### Dorcadion unidiscale

Taxon classificationAnimaliaColeopteraCerambycidae

Danilevsky, 1996 *

Figs 7K, L , 14E

###### Material examined.

Almaty Region: 10 km S of Kaskelen [Каскелен] (43°05'N, 76°35'E), 1735 m a.s.l., 13 V 2017, 15♂♂, 4♀♀, leg. RP; 10♂♂, 4♀♀, leg. JH; 14♂♂, 1♀, leg. KL.

###### Remarks.

*Dorcadionunidiscale* is endemic to southeastern Kazakhstan and it is known based on only a single population that is distributed in the area of Kaskelen on the northern slopes of the Trans-Ili Mountains (Danilevsky 1999). According to Toropov and Milko (2013), this species inhabits midmontane meadows at altitudes of 1500–2000 m a.s.l. The larvae feed on the roots of various grass species. The imagines (Fig. 14E) are active from the beginning of May to the first half of June.

##### Eodorcadion carinatum carinatum

Taxon classificationAnimaliaColeopteraCerambycidae

(Fabricius, 1781)

Fig. 8G

###### Material examined.

East Kazakhstan Region: Verkhnie Tainty [Верхние Таинты] env., (49°24'N, 83°03'E), 879 m a.s.l., 18 VI 2017, 1♂, leg. WTS.

###### Remarks.

*Eodorcadioncarinatum* is distributed from the South Urals through South Siberia, northern Kazakhstan and Mongolia to the territory of northeastern China. Five subspecies have been described to date and a nominotypical form occupies the western part of the species range (up to Krasnoyarsk). The species inhabits steppe and semi-desert habitats up to an altitude of 1900 m a.s.l. (Toporov and Milko 2013). The populations of all subspecies are usually characterised by a large number of individuals that occur on numerous plots. It seems to be primarily ecologically associated with *Agropyron* spp. and *Elmynus* spp. (Danilevsky 2007c). In the western part of its range, the larvae often feed on the roots of cereals and forage plant species (Plavilstshikov 1958). The adults start to appear in June and can be found until September (Danilevsky 2007c).

Only a single male that was hidden under a cow dung in a pasture habitat was collected (Fig. 14G).

##### Politodorcadion eurygyne eurygyne

Taxon classificationAnimaliaColeopteraCerambycidae

(Suvorov, 1911)

###### Material examined.

East Kazakhstan Region: 20 km NW of Tauke [Тауқе] (47°57'N, 83°16'E), 407 m a.s.l., 6 V 2017, 1♀, leg. RP.

###### Remarks.

*Politodorcadioneurygyne* is distributed in eastern Kazakhstan and West Siberia. This species includes two subspecies: *P.e.eurygyne* and *P.e.lailanum* Danilevsky, 2007 (Danilevsky 2018a). The nominotypical subspecies occurs in two separate regions in E Kazakhstan: north of Lake Zaysan and to the south of the Tarbagatay Mountains. Its range in the latter area is limited only to southern foothills of the Kalbinsky Ridge (Danilevsky 2007b). The beetles inhabit clayey deserts and semi-deserts (at an altitude of approx. 500 m a.s.l.) with sparse grass vegetation where they feed on various species of Poaceae (Toropov and Milko 2013).

##### Politodorcadion politum politum

Taxon classificationAnimaliaColeopteraCerambycidae

(Dalman, 1823)

Figs 8H, I , 14H

###### Material examined.

East Kazakhstan Region: 120 km NE of Ajagöz [Аягоз] (48°57'N, 80°55'E), 586 m a.s.l., 5 V 2017, 4♂♂, 1♀, leg. RP; 3♂♂, 1♀, leg. JH; 1♂, leg. GT; 125 km NE of Ajagöz [Аягоз] (48°57'N 80°54'E), 592 m a.s.l., 5 V 2017, 2♂♂, 1♀, leg. KL; 50 km S of Ajagöz [Аягоз] (47°37'N, 80°38'E), 747 m a.s.l., 8 V 2017, 1♂, leg. KL; 48 km N of Ajagöz [Аягоз] (48°22'N, 80°29'E), 727 m a.s.l., 5 V 2017, 2♂♂, leg. KL; 25 km E of Tarbagatay [Тарбагатай] (47°46'N, 81°36'E), 1128 m a.s.l., 8 V 2017, 1♂, 1♀, leg. RP; 3♂♂, leg. JH; 27 km E of Tarbabatay [Тарбагатай] (47°46'N, 81°36'E), 1119 m a.s.l., 8 V 2017, 7♂♂, 1♀, leg. KL.

###### Remarks.

*Politodorcadionpolitum* is distributed in northeastern Kazakhstan and southwestern Russia. The species includes three subspecies: *P.p.politum*, *P.p.akmolense* (Suvorov, 1911) and *P.p.shapovalovi* Danilevsky, 2006 (Danilevsky 2018a). The nominotypical subspecies is distributed west of Lake Zaysan and in West Siberia (Toropov and Milko 2013, Danilevsky 2018a, b). It inhabits grassy steppes that were mainly formed by *Stipa* spp. and sparse grasslands in hilly plains. The larvae feed on various species of Poaceae. The adults (Fig. 14H) are active from the end of April to mid-June (Toropov and Milko 2013).

##### Politodorcadion ribbei bobrovi

Taxon classificationAnimaliaColeopteraCerambycidae

(Danilevsky, 2001) *

Fig. 8J, K

###### Material examined.

East Kazakhstan Region: 12 km S of Zaysan [Зайсан] (47°21'N, 84°51'E), 965 m a.s.l., 7 V 2017, 1♂, 1♀, leg. RP; 1♂, 1♀, leg. KL.

###### Remarks.

This species is distributed in northeastern Kazakhstan and the Xinjiang region in China. The species includes two subspecies: *P.r.ribbei* (Kraatz, 1878) and *P.r.bobrovi*, whose populations are separated by the Manrak and Saur Mountain ranges (Toropov and Milko 2013, Danilevsky 2018a). *Politodorcadionribbeibobrovi* is endemic to Kazakhstan and occurs exclusively on the northern slopes of these mountains in the border zone with China. It inhabits clayey and stony semi-deserts in piedmont valleys with sparse grass vegetation. The larvae feed on various species of Poaceae. The adults are active from the end of April to the end of May (Toropov and Milko 2013).

#### Acanthocinini Blanchard, 1845

##### Exocentrus stierlini

Taxon classificationAnimaliaColeopteraCerambycidae

Ganglbauer, 1883

Fig. 4G, H

###### Material examined.

East Kazakhstan Region: Putintsevo [Путинцево] env. (49°52'N, 84°21'E), 472 m a.s.l., 19–23 VI 2017, 3♂♂, 2♀♀, leg. WTS; 1♂, leg. LK; 1♂, 1♀, (VI 2018 ex cult.) 2♂♂, from *Salix* sp., leg. MW; 2 exx., red wine trap, coll. LK & MB.

###### Remarks.

*Exocentrusstierlini* is an extremely rare but widespread species that is distributed from Central Europe to the Far East including northern Mongolia (Danilevsky 2014b, 2018a). Its occurrence in Mongolia was proven only very recently (Müller et al. 2013), similar to that of the Ulyanovsk region of Russia (Isaev and Ishutov 2001). According to Danilevsky (2014b), it is known to be monophagous on *Salix* within its entire range and there are no differences between specimens from Europe, Siberia and the Far East. The larvae develop in thin (6–22 mm diameter) willow shoots of both trees that are still alive or that are decaying. Pupation begins in May and continues until July. The adults occur in nature from June to August. Newly emerged imagines require supplementary feeding, which is conducted on the bark of young willow shoots. They lead a cryptic mode of life and can be found almost exclusively on their host plants (Cherepanov 1991a).

Although this species has already been mentioned as occurring in Kazakhstan (Danilevsky 2018a), this record was based only on the assumption concerning a single specimen from Staroaleyskoye (Altai Region of Russia, approx. 25 km from the Kazakh border) that is preserved in the collection of P. Svacha (Danilevsky 2014b, 2018, pers. comm.). However, until now, no specimens have been known directly from the borders of this country. Therefore, the presented locality in the area of Putintsevo is the first record for Kazakhstan.

Several specimens were beaten down from both live and dead willows of different ages, during hot and sultry weather in a deciduous forest that extends along the Khamir River in the foothills of the West Altai Mountains (Fig. 15D). The imagines of *E.stierlini* were collected in a few different habitats within one area in the Putinsevo environs. In addition to the rather shady and humid forest dominated by *Populus* and *Salix* where most of the specimens were found, beetles were also observed on an exposed site next to a river that had an admixture of *Betula* as well as in a more open habitat of a rather old *Populus* forest (Fig. 15F). At the last plot, two specimens were found in red wine traps. This is a rather peculiar observation concerning the representatives of the subfamily Lamiinae; however, the traps were hung in an air corridor, hence, they might have served as a mechanical barrier. Nevertheless, both individuals were caught on different days. Two mating couples were also observed. Only ten specimens were collected despite conducting many hours of targeted investigation over a few days during a rather optimal period, which underlines the rarity of this species. Attempting to attract them to an artificial light source, even at the site where the imagines were collected, did not provide the expected results. *Exocentrusstierlini* occurred sympatrically with other Lamiinae species, such as *Saperdasimilis*, *Lamiatextor* and *Rhopaloscelisunifasciatus*.

#### Mesosini Mulsant, 1839

##### Mesosa myops

Taxon classificationAnimaliaColeopteraCerambycidae

(Dalman, 1817)

###### Material examined.

East Kazakhstan Region: Putintsevo [Путинцево] env. (49°52'N, 84°21'E), 472 m a.s.l., 21–22 VI 2017, 1♂, 1♀, leg. WTS; 1♂, 2♀♀, (IV 2018 ex cult.) 1♂, from *Salix* sp., leg. LK; 1♀, leg. MW; Bykovo [Быково] env. (49°39'N, 84°33'E), 570 m a.s.l., 24 VI 2017, 1♂, 3 larvae, (24 VII 2017 ex larva) 1♂, from *Populus* sp., leg. MW.

###### Remarks.

This species is distributed from Eastern Europe through Siberia, including northern Kazakhstan, Mongolia and China, to the Far East and Sakhalin (Cherepanov 1990c, Danilevsky 2018a). It was widely discussed in a previous paper concerning the longhorn beetles of Mongolia (Karpiński et al. 2018).

The imagines were collected on the bark or beaten down from the dead branches and boughs of a few deciduous tree species (mostly of middle-aged birches *Betula*). Two specimens were additionally reared from the collected wood material of a fallen poplar *Populus* trunk and a thin willow *Salix* trunk.

#### Lamiini Latreille, 1825

##### Lamia textor

Taxon classificationAnimaliaColeopteraCerambycidae

(Linnaeus, 1758)

###### Material examined.

East Kazakhstan Region: Putintsevo [Путинцево] env. (49°52'N, 84°21'E), 472 m a.s.l., 22 VI 2017, 1♀, leg. MB, coll. MW.

###### Remarks.

*Lamiatextor* is a typical Palaearctic species that is distributed from Spain to Japan, including the Caucasus, Iran and Turkey (Danilevskaya et al. 2009). The larvae develop at the basal part of trunks and in the roots of various deciduous tree species, mainly of the poplars *Populus* and willows *Salix*.

A single female was beaten down from a young willow trunk in the habitat of a mountain deciduous forest.

##### Monochamus sartor urussovii

Taxon classificationAnimaliaColeopteraCerambycidae

(Fischer von Waldheim, 1805)

###### Material examined.

East Kazakhstan Region: Putintsevo [Путинцево] env. (49°52'N, 84°21'E), 472 m a.s.l., 21 VI 2017, 1♂, leg. WTS.

###### Remarks.

This taxon is widespread in Siberia and is distributed from Scandinavia and Eastern Europe (NE Poland) to the Far East and Japan (Plewa et al. 2018). It was discussed in a previous paper concerning the longhorn beetles of Mongolia (Karpiński et al. 2018). The taxonomic status of this species was uncertain. Wallin et al. (2013) considered *M.urussovii* to be a subspecies of *Monochamussartor* (Fabricius, 1787). This status was recently confirmed also by Plewa et al. (2018) by using different sets of data, such as morphology, genetics and ecology.

#### Saperdini Mulsant, 1839

##### Oberea kostini

Taxon classificationAnimaliaColeopteraCerambycidae

Danilevsky, 1988

Fig. 5F, G

###### Material examined.

East Kazakhstan Region: Putintsevo [Путинцево] env. (49°52'N, 84°21'E), 472 m a.s.l., 22 VI 2017, 1 ex., obs. WTS; 10 km S of Bayash Utepov [Баяш Утепов] (49°35'N, 82°28'E), 508 m a.s.l., 25 VI 2017, 1♂, 3♀♀, leg. WTS; 1♀, leg. MW; 1♀, leg. MB, coll. LK.

###### Remarks.

This is a locally occurring species that is distributed from the eastern part of European Russia to West Siberia and eastern Kazakhstan. The species is ecologically associated with the genus *Lonicera*. The larvae probably develop in the wood of living twigs and thin stems (Danilevskaya et al. 2009). According to Danilevsky (1988) and Yanovsky (2003), the adults are active in June and July.

Numerous specimens were collected in these two localities (Putintsevo and Sibinka River valley) in June 2005 (Danilevskaya et al. 2009).

In our research, only several rather old and damaged specimens were collected with a predominance of females, which may indicate the end of the appearance of this species. On the other hand, the imagines were rather active, flying around the host plants and sitting on the leaves only from time to time. On the first plot, this species inhabits *Lonicera* shrubs that border a river and a forest stand dominated by *Betula*, *Populus* and *Salix*. In the Sibinka River valley, the population of *O.kostini* develops in the shrubs that are growing on river banks as well as on stony areas around the valley (Fig. 15A).

##### Oberea ruficeps ruficeps

Taxon classificationAnimaliaColeopteraCerambycidae

Fischer von Waldheim, 1842 

Fig. 5H, I

###### Material examined.

Kyzylorda Region: Tartogay env. [Тартогай] (44°25'N, 66°13'E), 135 m a.s.l., 7 VI 2017, 1♂, leg. WTS; Almaty Region: 25 km SW of Kalinino [Басши] (43°53'N, 78°34'E), 691 m a.s.l., 13 VI 2017, 1♀, leg. MB, coll. LK.

###### Remarks.

The nominotypical subspecies is distributed in Kyrgizstan, Kazakhstan, Uzbekistan, western Siberia and northwestern China. The second subspecies – *O.ruficepsmuchei* Breuning, 1981 – is only known from Tajikistan (Danilevsky 2018a). The larvae probably develop in the stems and roots of plants of the genus *Euphorbia*.

This is a rarely collected species in Kazakhstan, where it is usually observed in tugay habitats. Ishkov and Kadyrbekov (2004) recorded this taxon, *inter alia*, in the Karatal and Ili River valleys.

In the environs of Tartogay, *O.ruficeps* was observed on a rather dry and salty bank of the Syr Darya River, which was mostly overgrown by *Elaeagnus*, *Tamarix* and *Halimodendron* (Fig. 10D). In the second locality, the species was found in a rather humid habitat near a small steam. The imagines were observed in flight in both cases; however, any *Euphorbia* species were not noticed on these plots.

##### Phytoecia coerulescens

Taxon classificationAnimaliaColeopteraCerambycidae

(Scopoli, 1763)

###### Material examined.

Almaty Region: 7 km W of Kabanbay [Қабaнбaй] (45°48'N, 80°31'E), 720 m a.s.l., 9 V 2017, 1♂, leg. RP; East Kazakhstan Region: Putintsevo [Путинцево] env. (49°52'N, 84°21'E), 472 m a.s.l., 21 VI 2017, 1♂, leg. WTS.

##### Phytoecia rufipes rufipes

Taxon classificationAnimaliaColeopteraCerambycidae

(Olivier, 1795)

###### Material examined.

Almaty Region: Kaskeleng [Каскелен] (43°12'N, 76°38'E), 825 m a.s.l., 13 V 2017, 1♀, leg. TJ, coll. RP.

###### Remarks.

This taxon is distributed from Southern Europe through North Africa, Asia Minor, the Caucasus and the Near East to Central Asia and South Siberia. The second subspecies – *P.rufipeslatior* Pic, 1895 – is only known from some regions in Syria and Turkey (Danilevsky 2018a). *Phytoeciarufipes* is an oligophagous species whose larvae develop in the roots of various herbaceous plants, particularly in *Foeniculumvulgare*, *Ferulagalbanifera* and other Apiaceae. The adults can be found on their host plants from May to July (Bense 1995).

##### Phytoecia nigricornis

Taxon classificationAnimaliaColeopteraCerambycidae

(Fabricius, 1782)

###### Material examined.

East Kazakhstan Region: Putintsevo [Путинцево] env. (49°52'N, 84°21'E), 472 m a.s.l., 23 VI 2017, 1♂, leg. MW.

##### Menesia sulphurata

Taxon classificationAnimaliaColeopteraCerambycidae

(Gebler, 1825)

###### Material examined.

East Kazakhstan Region: Bykovo [Быково] env. (49°39'N, 84°33'E), 571 m a.s.l., 21 VI 2017, 1 ex., obs. WTS.

###### Remarks.

This species is distributed from the eastern part of European Russia to the Far East and the Korean peninsula (Danilevsky 2018a). The larvae develop in the small diameter shoots and twigs of various deciduous trees. Its life cycle lasts from one to two years. The imagines fly from June to August (Cherepanov 1991b).

A single specimen was observed on a leaf of a harvested poplar at the edge of mountain deciduous grove consisted mainly of *Populus*, *Betula* and *Salix* (Fig. 15C).

##### Saperda alberti

Taxon classificationAnimaliaColeopteraCerambycidae

Plavilstshikov, 1915

Fig. 4I

###### Material examined.

East Kazakhstan Region: Putintsevo [Путинцево] env. (49°52'N, 84°21'E), 472 m a.s.l., 21 VI 2017, 2♀♀, leg. WTS.

###### Remarks.

*Saperdaalberti* is distributed from western Siberia to Japan, reaching the maximum of abundance in the Russian Far East (Danilevskaya et al. 2009). It was discussed in a previous paper concerning the longhorn beetles of Mongolia (Karpiński et al. 2018).

This species was recently published as a new for the Kazakh fauna by Danilevskaya et al. (2009) based on numerous specimens collected on *Populus* bark in the Putintsevo environs.

Two females were collected on the bark of a fresh poplar windfall on a river bank (Fig. 15E).

##### Saperda perforata

Taxon classificationAnimaliaColeopteraCerambycidae

(Pallas, 1773)

Fig. 4J

###### Material examined.

East Kazakhstan Region: Putintsevo [Путинцево] env. (49°52'N, 84°21'E), 472 m a.s.l., 20–21 VI 2017, 2♀♀, leg. WTS; 1♂, 1♀, leg. LK; Bykovo [Быково] env. (49°39'N, 84°33'E), 571 m a.s.l., 21 VI 2017, 4♂♂, 4♀♀, leg. WTS; 2♂♂, 4♀♀, leg. LK; 4♂♂, 5♀♀, leg. MW; 1♀, leg. MB.

###### Remarks.

This is a widespread species that is distributed from western Europe to the Far East, including North Africa and the Near East (Danilevsky 2018a). The larvae develop under the bark of deciduous trees, but they usually choose the poplar *Populus* and the willow *Salix* (Danilevskaya et al. 2009).

Numerous specimens were collected on the bark of harvested poplars, while some of them were attracted to artificial light sources (Fig. 15H).

##### Saperda scalaris

Taxon classificationAnimaliaColeopteraCerambycidae

(Linnaeus, 1758)

Fig. 4K

###### Material examined.

East Kazakhstan Region: Bykovo [Быково] env. (49°39'N, 84°33'E), 571 m a.s.l., 21 VI 2017, 1♀, leg. WTS.

###### Remarks.

This species is widespread in the Palaearctic region and is distributed from Western Europe to the Far East, while *S.s.hieroglyphica* (Pallas, 1773) ranges from European Russia through Kazakhstan, Mongolia and China to the Far East (Danilevsky 2018a).

In contrast to the nominotypical subspecies whose pubescence is intensively yellowish, this taxon is characterised by a constant bluish colour of its pale pubescence (Danilevsky 2018c). However, distinguishing a subspecies based only on a colour difference is rather doubtful, and therefore, it is considered by some authors (e.g. Sama 2002) to be a synonym of a nominotypical subspecies. According to Bussler (2013), both forms can be found in the Southern Carpathians. In this case, the colour variation may be caused by its association with a different host plant. In Siberia, this polyphagous species is mainly associated with the birch *Betulaplatyphylla* (Cherepanov 1991b), thus a rather whitish colouration may facilitate its camouflage on birch bark. Such a hypothesis seems to be confirmed by Hoskovec et al. (2016) in the case of *S.perforata*. The authors explained this phenomenon as a *prototypical mimicry* and claimed that the colour of imagines is determined by the host plant, and thus adults whose larvae developed in the Eurasian aspen *Populustremula* usually have a yellow-green or yellow-grey colour integument, whereas the beetles that developed in the white poplar *Populusalba* are usually grey. In Kazakhstan, we also observed similar white pubescence forms in the case of other related, though not associated with birches, species – *S.alberti*, *S.perforata* and *S.similis* (Fig. 4I, J, L). Therefore, a new synonymy is proposed: *Cerambyxscalaris* Linnaeus, 1758 = *Cerambyxhieroglyphicus* Pallas, 1773, syn. n.

A single female was found on the bark of a harvested birch log at the edge of mountain deciduous grove consisted mainly of *Populus*, *Betula* and *Salix* (Fig. 15C).

##### Saperda similis

Taxon classificationAnimaliaColeopteraCerambycidae

Laicharting, 1784

Fig. 4L

###### Material examined.

East Kazakhstan Region: Putintsevo [Путинцево] env. (49°52'N, 84°21'E), 472 m a.s.l., 22 VI 2017, 1♀, leg. MB, coll. MW.

###### Remarks.

This is a rather rare but widespread species that is distributed from eastern Europe to the Far East (Danilevsky 2018a). It was discussed in a previous paper concerning the longhorn beetles of Mongolia (Karpiński et al. 2018).

A single female was beaten down from a dead willow in a mountain deciduous forest dominated by *Populus* and *Salix*.

The collected specimen represents the white pubescence form (var. albopubescens Pic, 1925), which is characteristic for central-east Asia but rather rare in Europe (e.g. the Czech Republic, France). Very similar specimens are deposited in Abdysalom Kadyrov’s collection (Dushanbe) that represents cerambycid material from Tajikistan.

**Figure 1.**
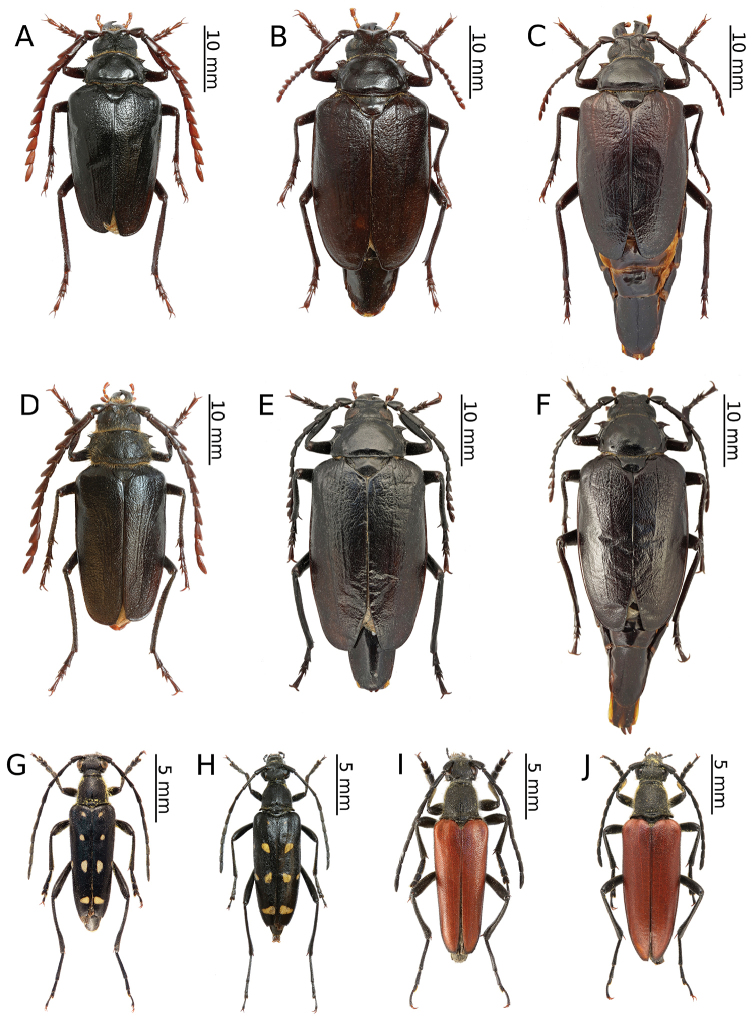
Photos of longhorn beetles specimens collected during the expedition to Kazakhstan in 2017: **A***Psilotarsusbrachypterusbrachypterus* (male) **B***P.brachypterusbrachypterus* (female) **C***P.brachypterusbrachypterus* (female, abdomen filled with eggs) **D***P.brachypteruspubiventris* (male) **E***P.brachypteruspubiventris* (female) **F***P.brachypteruspubiventris* (female, abdomen filled with eggs) **G***Lepturaduodecimguttata* (male) **H***L.duodecimguttata* (female) **I***Lepturalianigripesrufipennis* (male) **J***L.nigripesrufipennis* (female).

**Figure 2.**
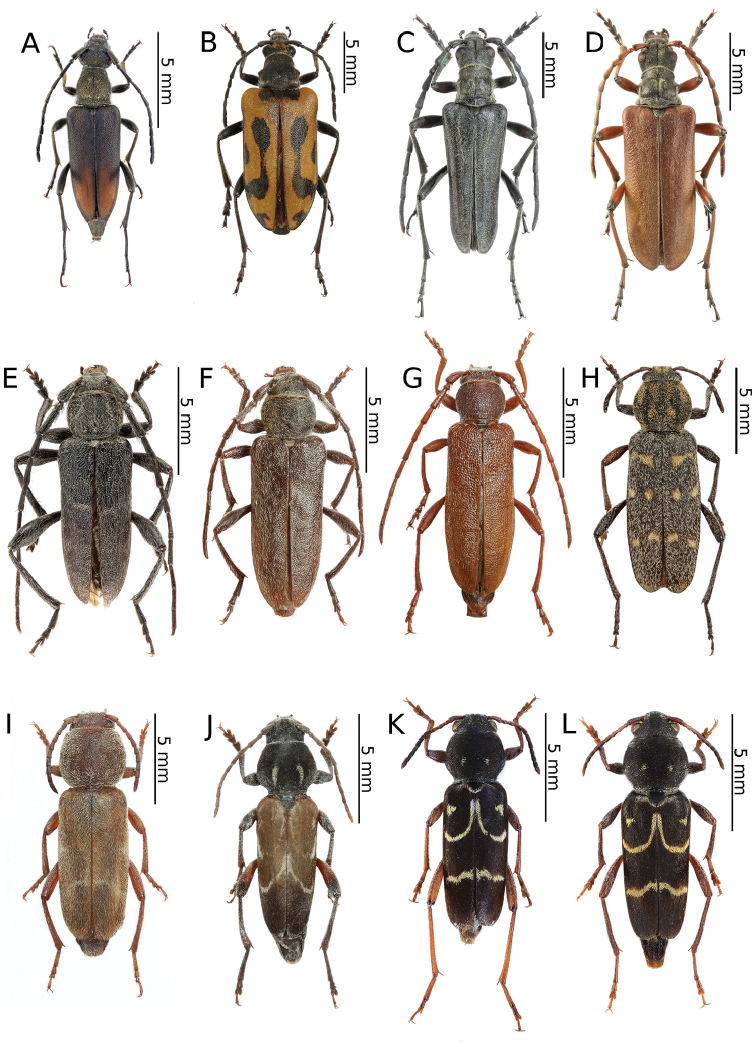
Photos of longhorn beetles specimens collected during the expedition to Kazakhstan in 2017: **A***Anastrangaliasequensi* (female, melanistic form) **B***Brachytainterrogationisrussica* (female) **C***Stenocorusminutus* (male) **D***S.minutus* (female) **E***Turaniumscabrum* (male, dark form) **F***T.scabrum* (female, dark form) **G***T.scabrum* (female, light form) **H***Xylotrechusadspersus* (male) **I***Xylotrechusalakolensis* (male) **J***Xylotrechushircus* (female) **K***Xylotrechuscapricornus* (male) **L***X.capricornus* (female).

**Figure 3.**
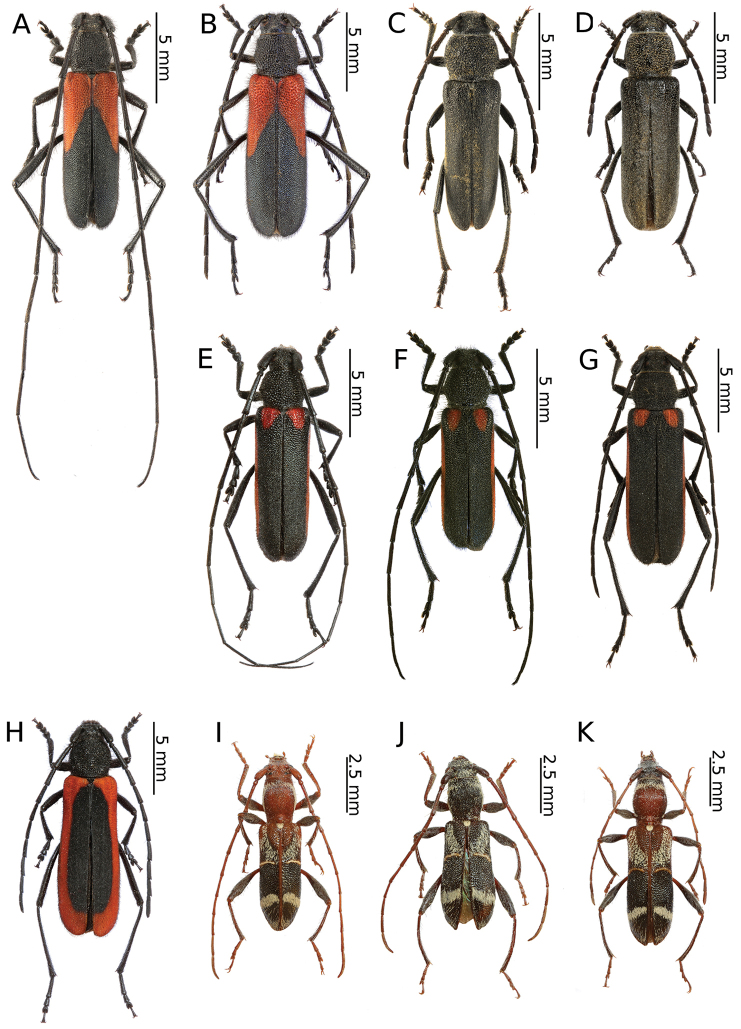
Photos of longhorn beetles specimens collected during the expedition to Kazakhstan in 2017: **A***Anoplistesjacobsoni* (male) **B***A.jacobsoni* (female) **C***Anoplistesgalusoi* (male) **D***A.galusoi* (female) **E***Anoplisteshalodendrihalodendri* (male, Sibinka River valley) **F***A.halodendrihalodendri* (male, Tarbagatay environs) **G***A.halodendrihalodendri* (female, Sibinka River valley) **H***Amarysiusduplicatus* (female) **I***Cleroclytussemirufuscollaris* (male, light form) **J***C.semirufuscollaris* (male, dark form) **K***C.semirufuscollaris* (female, light form).

**Figure 4.**
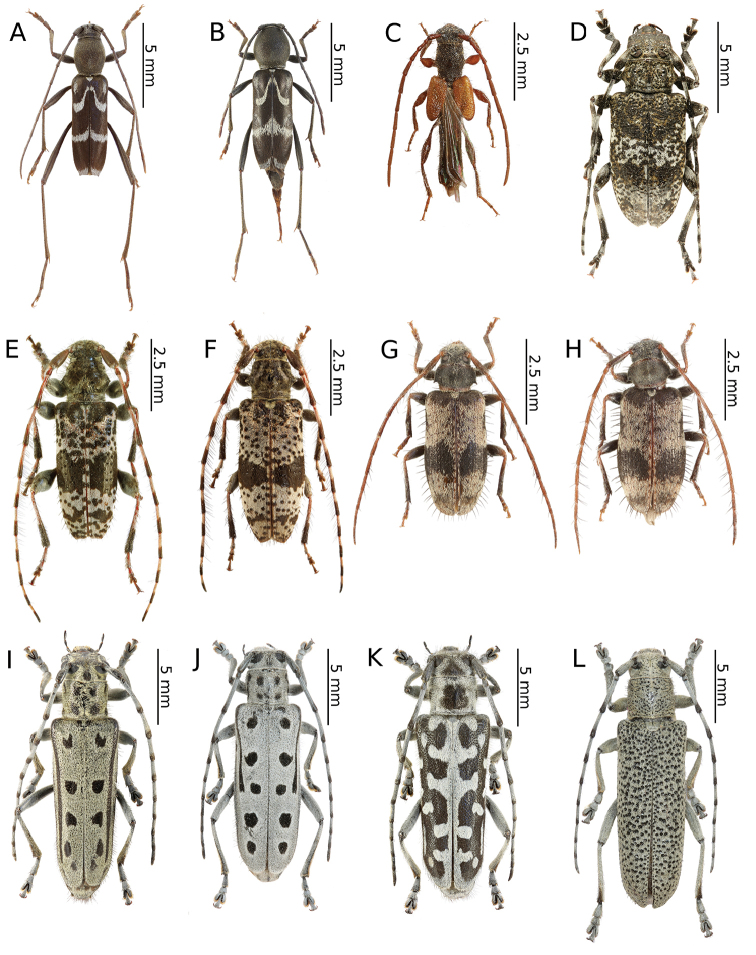
Photos of longhorn beetles specimens collected during the expedition to Kazakhstan in 2017: **A***Rhaphumagracilipes* (male) **B***R.gracilipes* (female) **C***Molorchusschmidti* (female) **D***Aegomorphusobscurior* (female) **E***Ropaloscelisunifasciatus* (male) **F***R.unifasciatus* (female) **G***Exocentrusstierlini* (male) **H***E.stierlini* (female) **I***Saperdaalberti* (female, whitish form) **J***Saperdaperforata* (female, whitish form) **K***Saperdascalaris* (female, whitish form) **L***Saperdasimilis* (female, whitish form).

**Figure 5.**
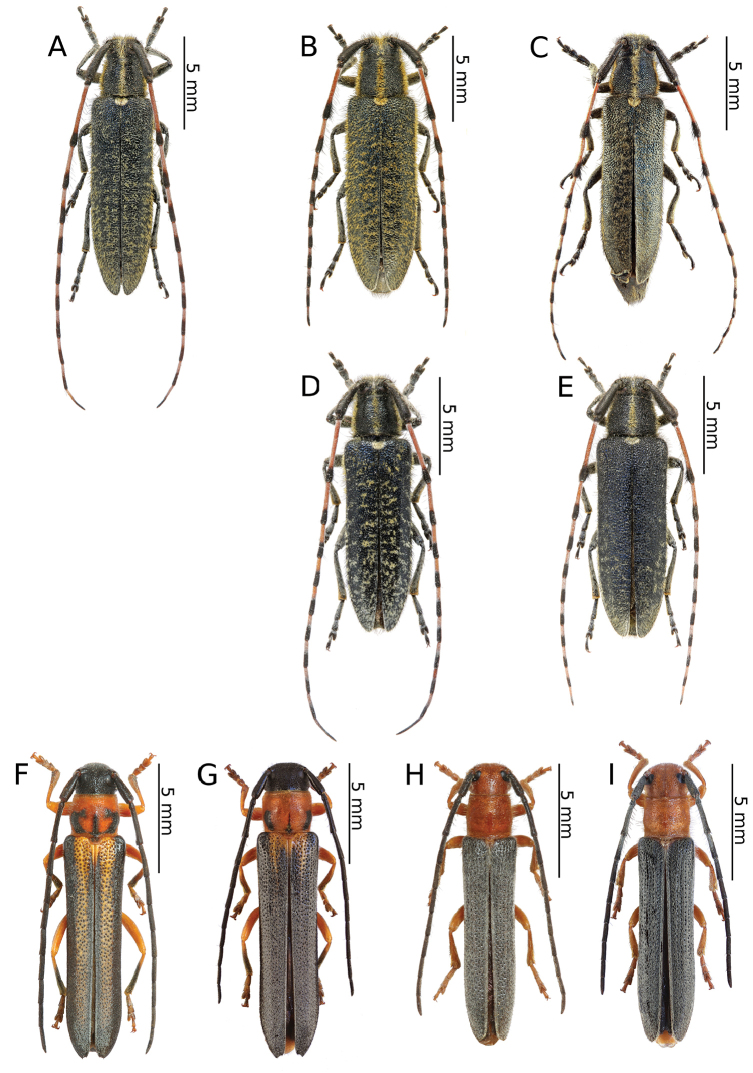
Photos of longhorn beetles specimens collected during the expedition to Kazakhstan in 2017: **A***Agapanthiadahlicalculensis* (male) **B***A.dahlicalculensis* (female) **C***A.dahli* spp. (female, Karashota environs) **D***Agapanthiaalternansalternans* (male) **E***A.alternansalternans* (female) **F***Obereakostini* (male) **G***O.kostini* (female) **H***Oberearuficepsruficeps* (male) **I***O.ruficepsruficeps* (female).

**Figure 6.**
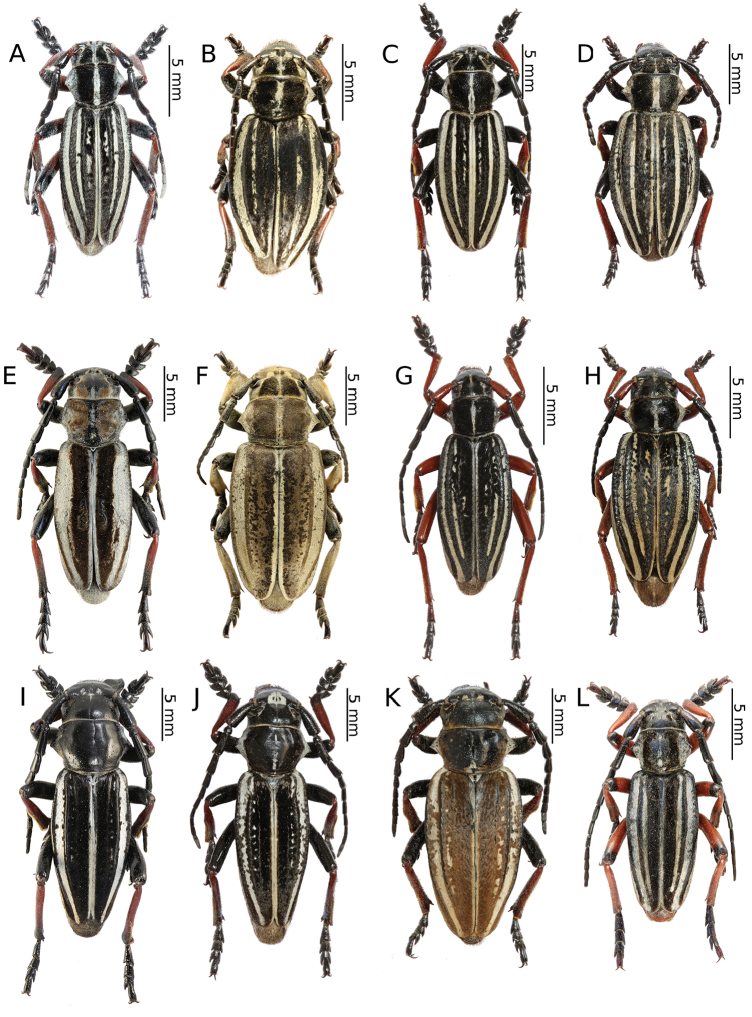
Photos of longhorn beetles specimens collected during the expedition to Kazakhstan in 2017: **A***Dorcadionabakumovisarkandicum* (male) **B***D.abakumovisarkandicum* (female) **C***Dorcadiontenuelineatum* (male) **D***D.tenuelineatum* (female) **E***Dorcadioncrassipescrassipes* (male) **F***D.crassipescrassipes* (female) **G***Dorcadionacutispinum* (male) **H**D.acutispinum (female) **I***Dorcadiongeblerigebleri* (male, Zaysan environs) **J***D.geblerigebleri* (male, Kabanbay environs) **K***D.geblerigebleri* (female) **L***Dorcadionarietinumarietinum* (male).

**Figure 7.**
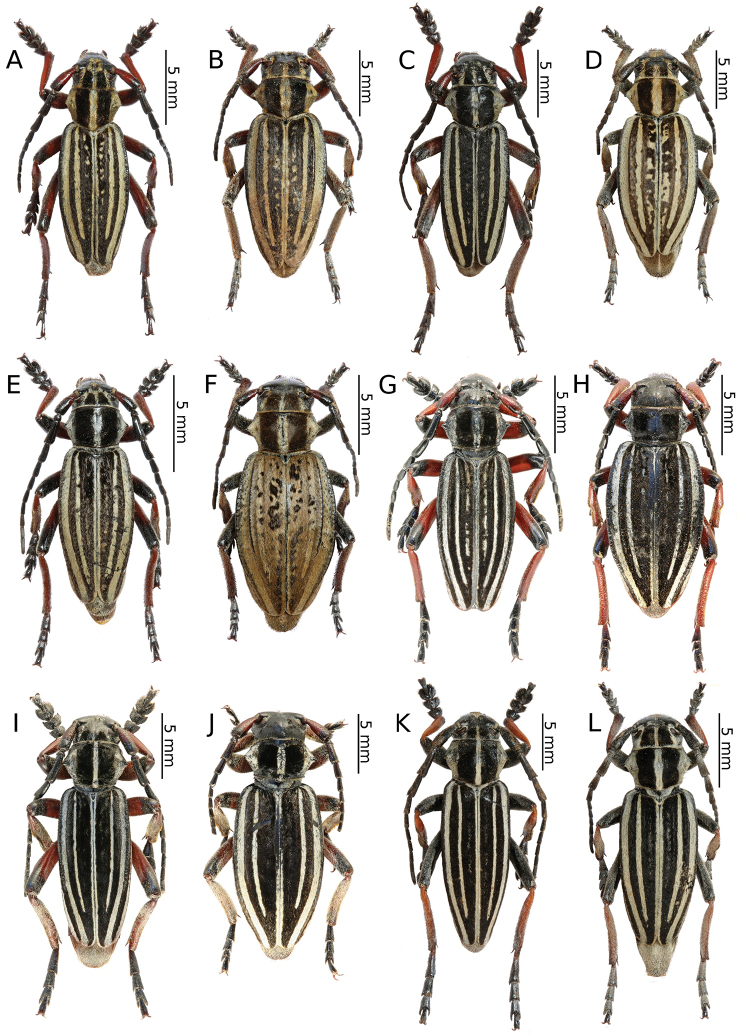
Photos of longhorn beetles specimens collected during the expedition to Kazakhstan in 2017: **A***Dorcadionabsinthiumishkovi* (male) **B***D.absinthiumishkovi* (female) **C***Dorcadionkapchagaicum* (male) **D***D.kapchagaicum* (female) **E***Dorcadionnikolaevi* (male) **F***D.nikolaevi* (female) **G***Dorcadionsuvorovikonyrolenum* (male) **H***D.suvorovikonyrolenum* (female) **I***Dorcadiontianshanskiiradkevitshi* (male) **J***D.tianshanskiiradkevitshi* (female) **K***Dorcadionunidiscale* (male) **L***D.unidiscale* (female).

**Figure 8.**
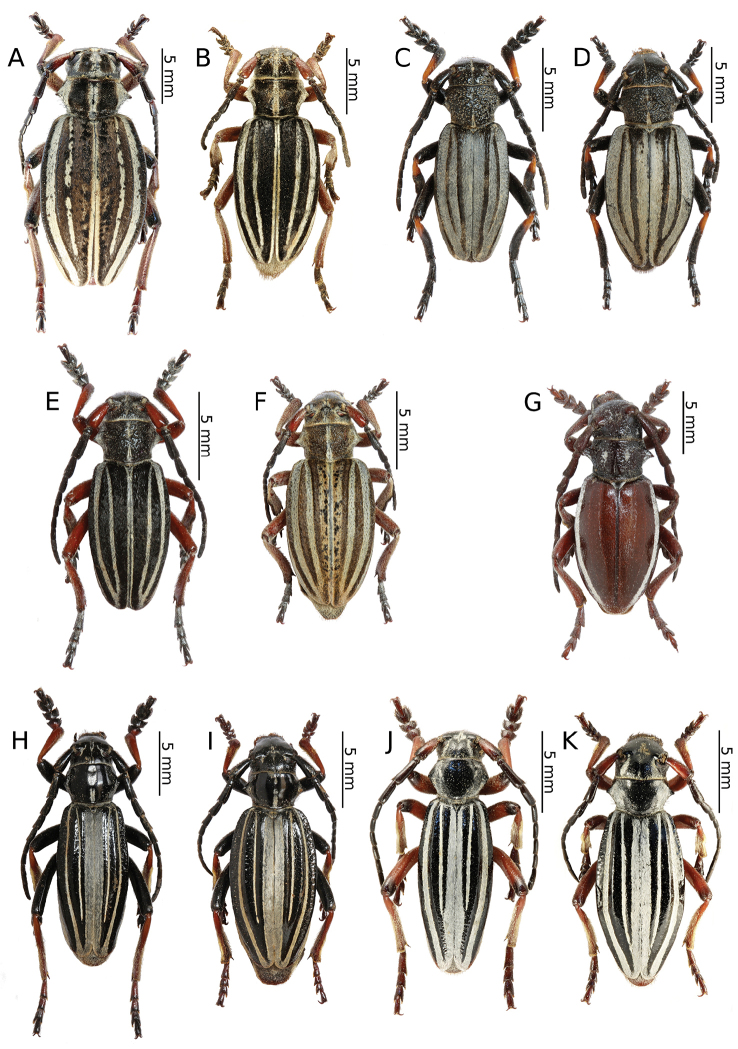
Photos of longhorn beetles specimens collected during the expedition to Kazakhstan in 2017: **A***Dorcadionsongaricum* (female) **B***Dorcadionmorozovi* (female) **C***Dorcadionsemenovisemenovi* (male) **D***D.semenovisemenovi* (female) **E***Dorcadionsokolowi* (male) **F***D.sokolowi* (female) **G***Eodorcadioncarinatumcarinatum* (male) **H***Politodorcadionpolitumpolitum* (male) **I***P.politumpolitum* (female) **J***Politodorcadionribbeibobrovi* (male) **K***P.ribbeibobrovi* (female).

**Figure 9.**
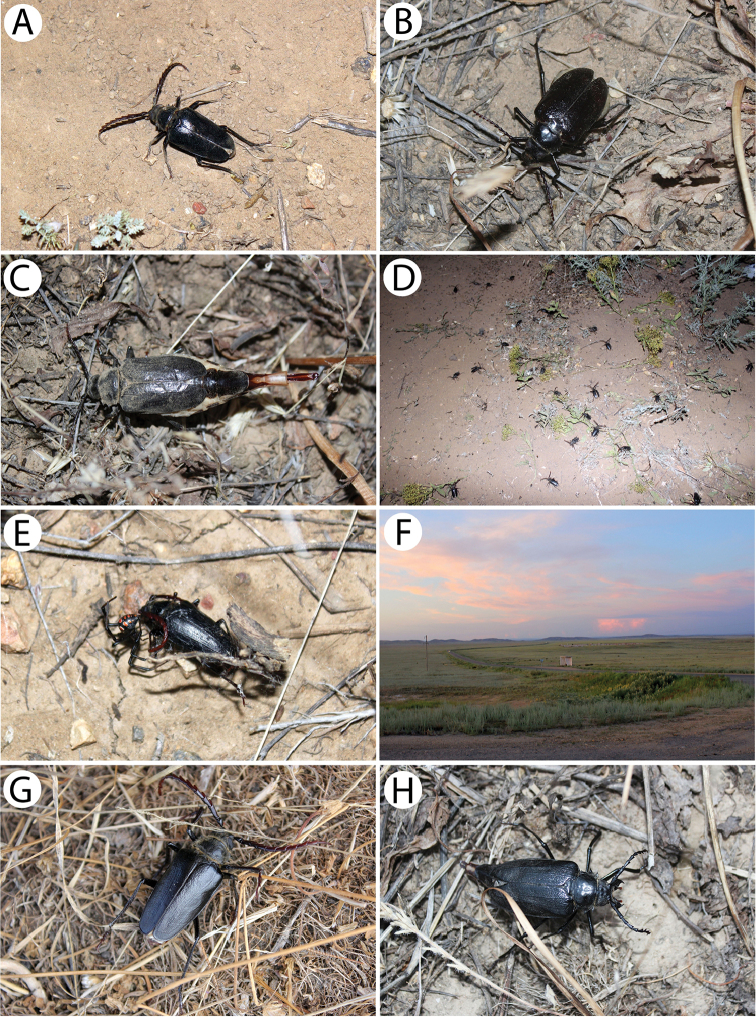
Field photos of imagines in nature and habitats of typical Kazakh cerambycid species: **A** male of *Psilotarsusbrachypterusbrachypterus***B** female of *P.brachypterusbrachypterus***C** female of *P.brachypterusbrachypterus* while spraying pheromones with raised ovipositor **D** massive occurrence of the males of *P.brachypterusbrachypterus* attracted to an artificial light source **E** male of *P.brachypterusbrachypterus* hunted by *Latrodectustredecimguttatus***F***Artemisia*-desert in Kurshim environs, the habitat of *P.brachypterusbrachypterus***G** male of *Psilotarsusbrachypteruspubiventris***H** female of *P.brachypteruspubiventris*.

**Figure 10.**
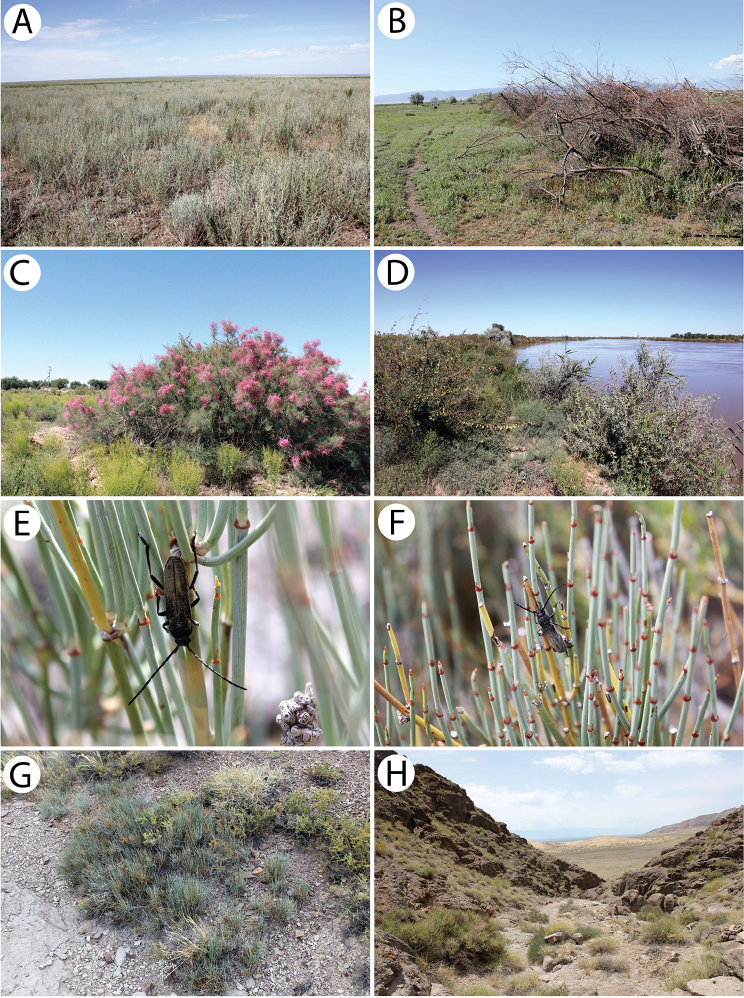
Field photos of imagines in nature, host plants and habitats of typical Kazakh cerambycid species: **A** semi-shrub desert in Kapchagay environs, the habitat of *Psilotarsusbrachypteruspubiventris***B** flood barrier formed with oleasters branches, the habitat of *Turaniumscabrum* and *Chlorophoruselaeagni***C** blossoming tamarisks in the habitat of *Ch.elaeagni* and *Anoplistesjacobsoni***D** bank of the Syr Darya River in Tartogay environs, the habitat of *Oberearuficepsruficeps***E** male of *Anoplistesgalusoi***F***A.galusoi* on *Ephedrastrobilacea***G** shrubs of *E.strobilacea*, the host plant of *A.galusoi***H** mountain slopes in Altyn-Emel National Park, the habitat of *A.galusoi*.

**Figure 11.**
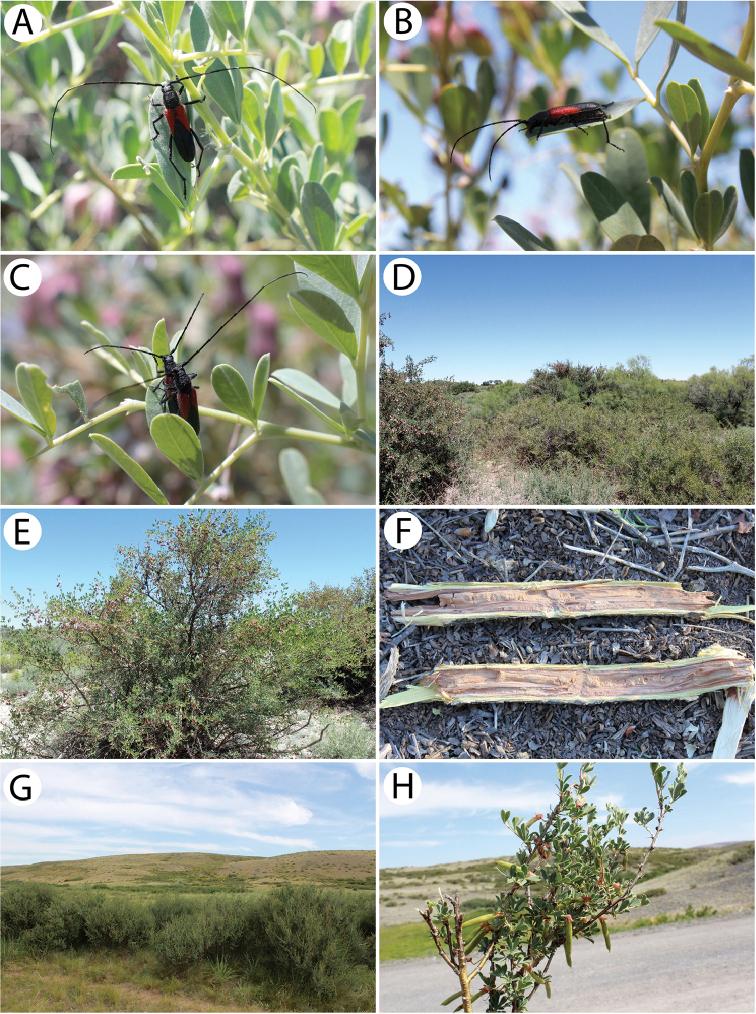
Field photos of imagines in nature, host plants and habitats of typical Kazakh cerambycid species: **A** male of *Anoplistesjacobsoni***B** female of *A.jacobsoni***C** pair of *A.jacobsoni in copula* on *Halimodendronhalodendron***D** tugays with *Halimodendron*, *Tamarix* and *Eleagnus* in Tartogay environs, the habitat of *A.jacobsoni* and *Chlorophoruselaeagni***E** shrub of *H.halodendron*, the host plant of *A.jacobsoni***F** larval feeding gallery of *A.jacobsoni* in a stem of *H.halodendron***G***Caragana* shrubs in Tarbagatay environs, the habitat of *Anoplisteshalodendrihalodendri***H***Caragana* sp., the host plant of *A.halodendrihalodendri*.

**Figure 12.**
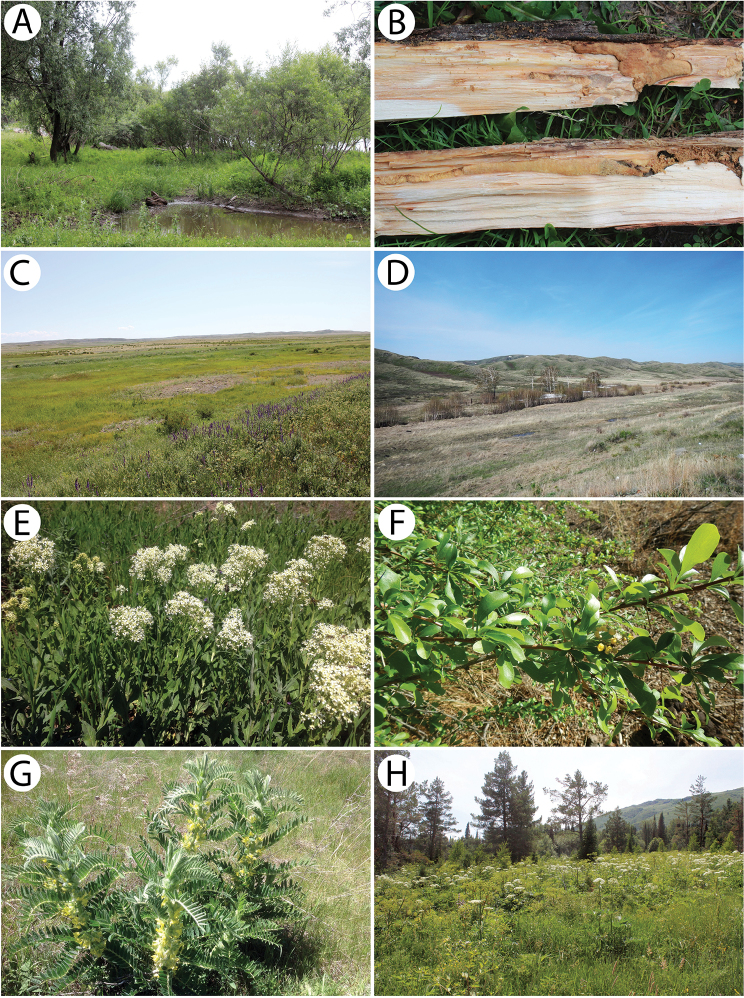
Field photos of host plants, larval feeding galleries and habitats of typical Kazakh cerambycid species: **A** river bank overgrown by willows, the habitat of *Xylotrechusadspersus***B** larval feeding gallery of *X.adspersus* in a branch of *Salix* sp. **C** roadside vegetation strip in Taskesken environs, the habitat of *Xylotrechusalakolensis***D** hilly grove with birches, the habitat of *Xylotrechushircus***E** inflorescences of *Lepidiumdraba*, the food plant of *Cleroclytussemirufuscollaris***F***Berberisvulgaris*, the host plant of *C.semirufuscollaris***G***Astragalussieversianus*, possible host plant of *Agapanthiaviolacea***H** roadside strip with herbaceous vegetation, the habitat of *Agapanthiaalternansalternans*.

**Figure 13.**
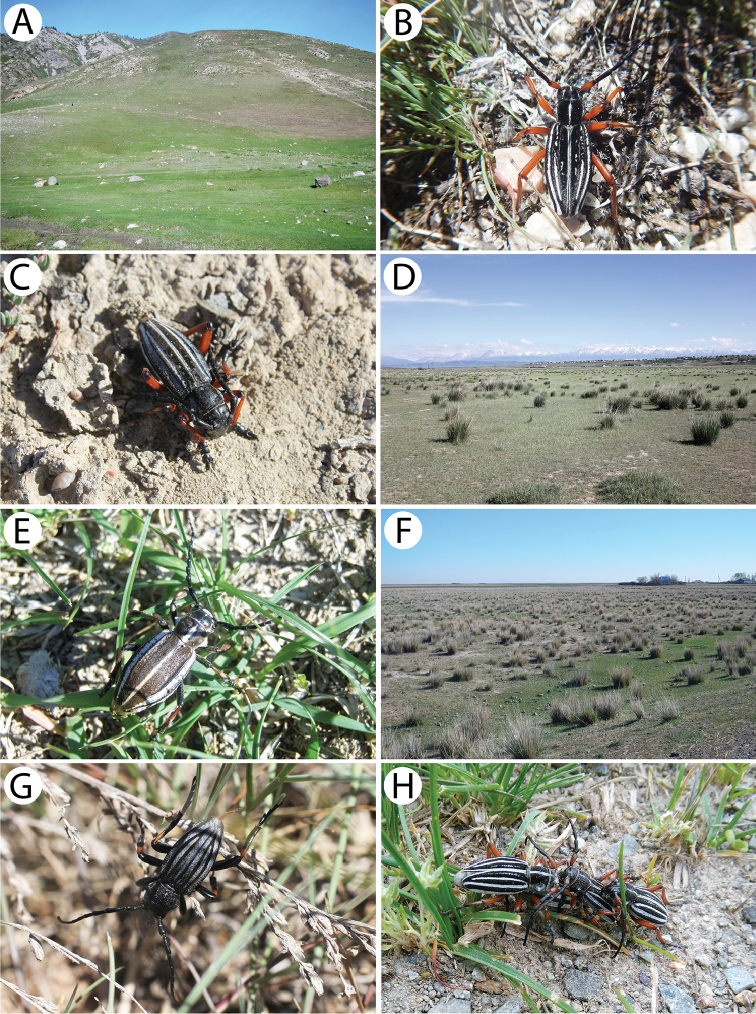
Field photos of imagines in nature and habitats of typical Kazakh cerambycid species: **A** steppe area in Arkhaly environs, the habitat of *Dorcadionabsinthiumishkovi***B** male of *Dorcadionacutispinum***C** male of *Dorcadionarietinumarietinum***D** mountain steppe area in Kegen environs, the habitat of *D.arietinumarietinum*, *Dorcadioncrassipescrassipes* and *Dorcadionsemenovisemenovi***E** female of *Dorcadiongeblerigebleri***F** mountain steppe area in Zaysan environs, the habitat of *D.geblerigebleri***G** male of *D.semenovisemenovi***H** particular behavior of a few individuals of *Dorcadionsokolowi*.

**Figure 14.**
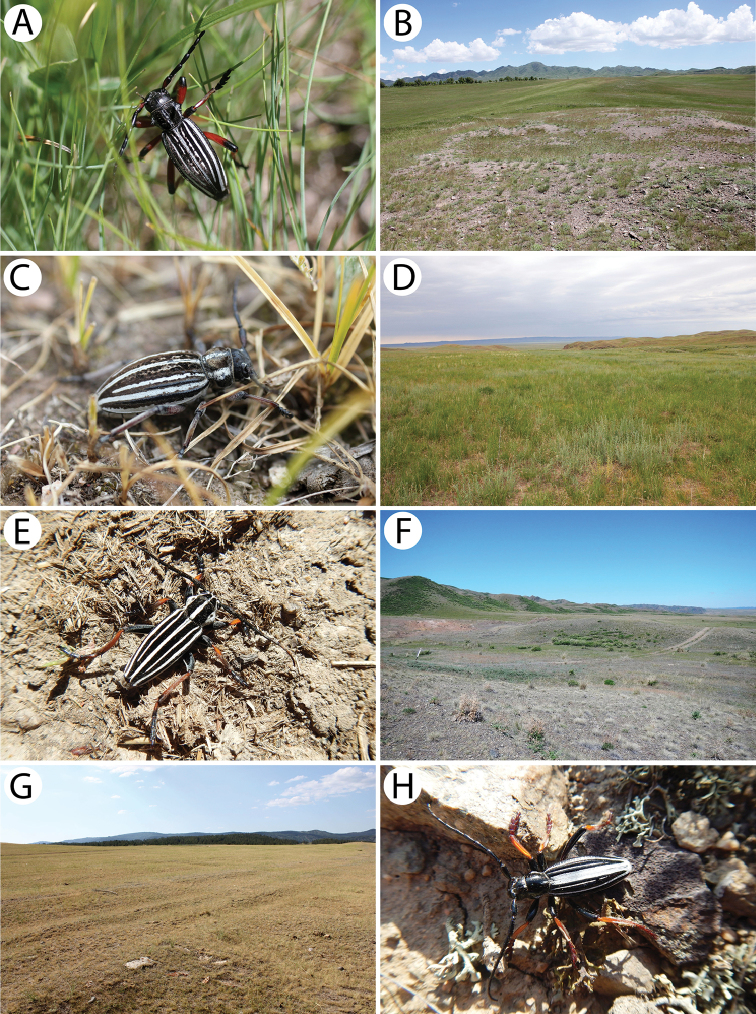
Field photos of imagines in nature and habitats of typical Kazakh cerambycid species: **A** female of *Dorcadionsuvorovikonyrolenum***B** steppe area in Karlygash environs, the habitat of *D.suvorovikonyrolenum***C** female of *Dorcadiontianshanskiiradkevitshi***D** pasture in Kenen environs, the habitat of *D.tianshanskiiradkevitshi***E** male of *Dorcadionunidiscale***F** mountain steppe area in Kyzyl Kesik environs, the habitat of *Dorcadionsongaricum***G** pasture in Verkhnie Tainty environs, the habitat of *Eodorcadioncarinatumcarinatum***H** male of *Politodorcadionpolitumpolitum*.

**Figure 15.**
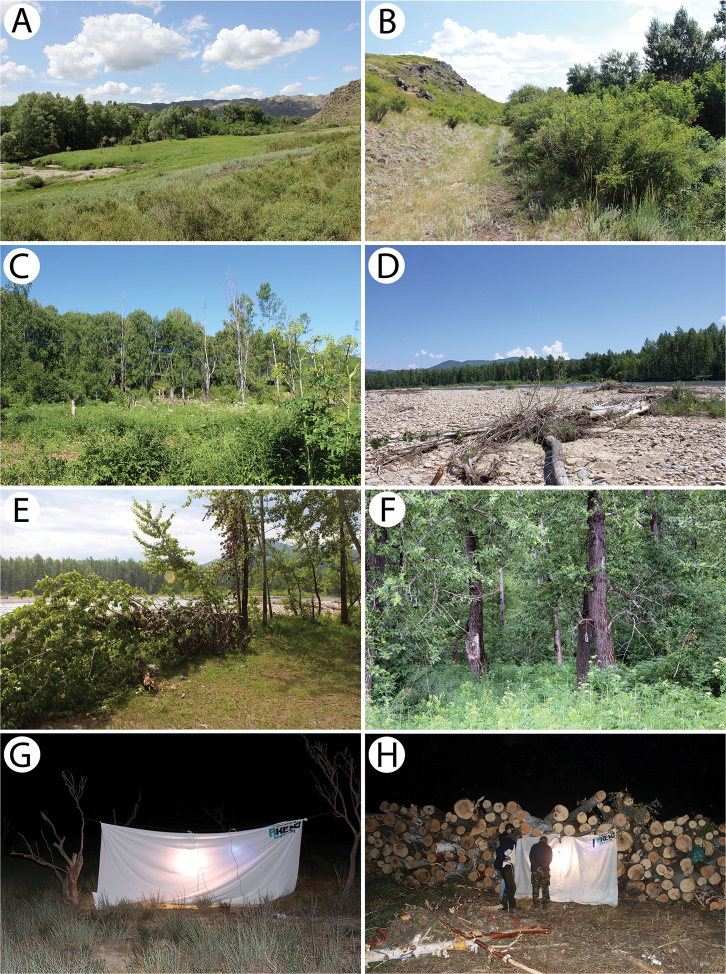
Field photos of habitats of typical Kazakh cerambycid species and some methods used: **A** general view on Sibinka River valley, the habitat of several collected species **B** pea shrubs on stony hills in Sibinka River valley, the habitat of *Stenocorusminutus* and *Anoplisteshalodendrihalodendri***C** mountain deciduous grove in Bykovo environs, the habitat of numerous collected species **D** general view on mountain deciduous forest along the Khamir River in Putinsevo environs, the habitat of numerous collected species **E** river bank with poplar windfalls, the habitat of *Saperdaalberti***F** wine trap in poplars forest, the habitat of *inter alia Macrolepturathoracica*, *Rhaphumagracilipes* and *Exocentrusstierlini***G** attracting insects to artificial light source in *Artemisia*-desert habitat **H** attracting insects to artificial light source at the edge of a mountain deciduous forest.

## Discussion

The flora of Kazakhstan amounts to more than 13 000 species, including approx. 5750 representatives of gymnosperms. As many as 14% of the total plant species are endemics in various degrees, many of them are additionally relicts. Moreover, Kazakhstan, due to its unique combination of natural complexes of steppes, deserts and mountain ranges, which are connected with major inland water and river systems, provides a wide variety of habitats and relevant types of flora that are connected with the arid regions of Central Asia. Apart from two centres of the endemism of the flora (the Karatau Mountains and the Western Tien Shan), there are many unique natural ecosystems, such as desert communities in Betpak-Dala, xylium, shrub and steppe communities of the Southern Altai, spruce and apple forests in the foothills and mountains of Dzungarian Alatau and Tien Shan and floodplain (riparian) forests in the Syr Darya and Ili River valleys (MEWR 2014).

This huge variety of habitats as well as the presence of many endemic plant species creates a unique diversity of invertebrates, also among the representatives of Coleoptera. According to the records of the State Forest Fund of the Republic of Kazakhstan, afforested areas cover only 4.61% of the total territory, and they consist of more than 20 tree and 40 shrub species. The forests in Kazakhstan can be divided into several types including pine forests in the Kazakh uplands, mountain forests in the Altai, Saura, Dzungarian Alatau and the Tien Shan Mountains, saxaul forests and riparian intrazonal forests (MEWR 2014). Special attention should be paid to the unique forest enclave on the western slopes of the Altai Mountains in the northeastern part of the country. According to Danilevskaya et al. (2009), northeastern Kazakhstan is generally a very interesting area from a zoogeographical point of view. In this place, the easternmost localities of European species are situated next to the westernmost sites of East Asian taxa, which creates the high-level biodiversity of the region. That was also confirmed in the case of representatives of the family Cerambycidae.

A total of 78 species belonging to the subfamilies of Prioninae, Lepturinae, Cerambycinae and Lamiinae was recorded as a result of the field research conducted during our two expeditions. They represent approx. 30% of the cerambycid fauna that is known from the entire country’s area. Three species – *Psilotarsusbrachypterus* (Gebler, 1830), *Dorcadionarietinum* Jakovlev, 1898 and *D.gebleri* Kraatz, 1873 – were represented by two different subspecies. Among 81 taxa that were collected, as many as 19 (approx. 23%), mainly in the genera of *Anoplistes* and *Dorcadion*, are endemic to Kazakhstan. The next 14 (approx. 18%) are endemic in regard to either the neighbouring region of Xinjiang (e.g. *Dorcadionmorozovi* Danilevsky, 1992, *D.sokolowi* Jakovlev, 1899), to the region of Central Asia – e.g. *Turaniumscabrum* (Kraatz, 1882), *Oberearuficepsruficeps* Fischer von Waldheim, 1842 – or to western Mongolia and West Siberia, e.g. *Stenocorusminutus* (Gebler, 1841). Some little-known species, such as *Anoplistesforticornis* Reitter, 1901, *A.diabolicus* Reitter, 1915 and *Turkaromiapruinosa* (Reitter, 1903), were not found despite conducting many hours of targeted investigation during a rather optimal period in suitable habitats or even on the exact plots that had been recorded by other authors. Although it may be related to phenological changes in different years, it also may indicate the declining of the particular populations of these species. According to Kadyrbekov et al. (1996), some cerambycids, such as *Hesperophanesheydeni* Baeckmann, 1923 and *Dorcadionbalchashense* (Suvorov, 1911), have reduced their ranges in last 30–40 years, while other, e.g. *Anoplistesgalusoi* Kostin, 1974, *T.pruinosa* and *Dorcadiongrande* Jakovlev, 1906, survived in small areas due to anthropogenic transformation of their habitats. The part of our research that was focused mainly on the saproxylic representatives of the family Cerambycidae was carried out in the area of Putintsevo from 19 to 23 June and resulted in finding 40 species. Another scientific expedition, which was also devoted to longhorn beetles, was conducted in this region in 2005 by M. Danilevsky’s research team (Danilevskaya et al. 2009). The authors recorded 59 species from 8 to 30 June. Since both surveys took place in the similar period the results can be compared to some extent. As many as 33 species, which constitute approx. 83% of taxa collected by us, were common in both studies. Most of the common species (in genera such as *Asemum*, *Euracmaeops*, *Gnathacmaeops*, *Pachyta*, *Molorchus* and *Pogonocherus*) that were not collected are ecologically associated with conifers and their lack in our research was caused by insufficient investigation of the areas with main share of *Abies*, *Picea*, *Larix* and *Pinus*. However, the lack or a very small number of individuals of some species that live on deciduous trees (e.g. *Amarysiusduplicatus* Tsherepanov, 1980 on *Spiraea* and *Obereakostini* Danilevsky, 1988 on *Lonicera*) is clearly connected to phenological changes. Some other species, *inter alia*, *Amarysiussanguinipennis* Blessing, 1872, *Xylotrechusibex* (Gebler, 1825) and *Necydalismajor* Linnaeus, 1758, are in turn very rare or lead the cryptic mode of life. On the other hand, a few interesting species, such as *Exocentrusstierlini* (Ganglbaur, 1883) and *Saperdasimilis* Laicharting, 1784, were not found in 2005. A similar comparision can be made for the locality in the Sibinka River valley. In 2005, the research team of M. Danilevsky found there 22 species including *Xylotrechusadspersus* (Gebler, 1830) and *Politodorcadioneurygyneeurygyne* (Suvorov, 1911), while only 8 taxa were collected by our group. However, an interesting and infrequent species – *Stenocorusminutus* – was found there only in 2017.

The greatest contributions to the knowledge of longhorn beetles from the area of Kazakhstan – particularly to the regions of the South and East – have been made by Kostin (1968a,b, 1973, 1974, 1978) and Kadyrbekov (1999, 2004), as well as in collaborations with other authors: Kadyrbekov, Childebaev and Yashchenko (1996), Kadyrbekov and Tleppaeva (1997, 2004a,b, 2016), Kadyrbekov, Ishkov and Tleppaeva (1998), Kadyrbekov, Tleppaeva and Childebaev (2003), Ishkov and Kadyrbekov (2004), Kadyrbekov and Childebaev (2007), Kadyrbekov, Tleppaeva and Mansurova (2010). However, the outstanding input concerning Cerambycidae of Central and Northern Asia, which comprises the region discussed here, has also been made by Plavilstshikov (1936, 1940, 1958), Cherepanov (1990a,b,c, 1991a,b), Danilevsky (e.g. 1996a,b,c, 1999, 2000, 2001a,b,c, 2002, 2004, 2007a,b,c, 2009, 2012, 2014a, 2017, 2018d), Lazarev (2011, 2013a,b, 2014) and Shapovalov (2014). Moreover, an interesting expedition to northeastern Kazakhstan was also conducted by M. Danilevsky’s research team in mid-June of 2005 (Danilevskaya et al. 2009). It was R. Kadyrbekov and related scientists who recorded and observed numerous rare and endemic taxa in the territory of South and East Kazakhstan. Among many others, *Microarthronkomaroffi* Dohrn, 1885, *Dokhtouroffianebulosa* Gebler, 1845, *Tetropiumstaudingeri* Pic, 1901, *Apatophysisserricornis* Gebler, 1843, *A.baeckmanniana* Semenov, 1907, *Phymatodeshauseri* Pic, 1907, *Hesperophanesheydeni*, *Molorchuspallidipennis* Heyden, 1887, *Xylotrechuszaisanicus* Plavilstshikov, 1940, *Anoplistesdiabolicus*, *A.galusoi* and *Dorcadionprofanifuga* Plavilstshikov, 1951 are particularly interesting.

Despite all of the aforementioned studies, the cerambycid fauna of Kazakhstan is still not sufficiently recognised. This is evidenced by the many new taxa that have been described from the southern and eastern parts of the country in the last several years. The new endemic genus *Murzinia* Lazarev, 2011, which is additionally represented by a rather large species – *M.karatauensis* Lazarev, 2011 – deserves special attention. Among the recently published species from the region are *Cortoderakokpektensis* Danilevsky, 2007, *Xylotrechusalakolensis* Karpiński & Szczepański, 2018, *X.katerinae* Shapovalov, 2014, *Agapanthiadanilevskyi* Lazarev, 2013 and *A.parauliensis* Danilevsky, 2017. Furthermore, many new subspecies, i.e. *Psilotarsusheydenialatauensis* Danilevsky, 2014, *Brachytavariabilisshapovalovi* Lazarev, 2014, *Stenocorusvalidicornismediocris* Danilevsky, 2012, *Xylotrechusarnoldiitenebrosus* Shapovalov, 2014, *Dorcadionpantherinumludmilae* Abramov, 2018, *Agapanthiaalternansparalternans* Danilevsky, 2017 and *Tetropselaeagnishapovalovi* Danilevsky, 2018, were discovered in this region only in the last few years. Additionally, some taxa that were already known may still be found here as new to the country. One example is *Exocentrusstierlini* recorded here for the first time from the area of Putintsevo. Five other cerambycid species, recently found in the same area, are new for Kazakhstan: *Euracmaeopssmaragdulus* Fabricius, 1793, *Amarysiussanguinipennis*, *A.duplicatus*, *Rhopaloscelisunifasciatus* Blessig, 1873 and *Saperdaalberti* Plavilstshikov, 1916. Although *A.duplicatus* was collected earlier by I. Kostin near Ust-Kamenogorsk in 1960, it was identified as *A.altajensis* Blessing, 1872, and the another finding in the same locality by A. Napolov in 1994 has not been published (Danilevskaya et al. 2009).

In addition to the taxonomic studies, the biology and ecology of the longhorn beetles that are distributed in this region should also be thoroughly investigated. More and more of the vulnerable Kazakh endemics are particularly interesting. The bionomy of many local species, for example in the genera of *Psilotarsus*, *Apatophysis*, *Anoplistes* and Xylotrechus (Kostiniclytus), requires further research. Herein we report on *Halimodendronhalodendron* as a host plant of *Anoplistesjacobsoni* Baeckmann, 1904, at the same time questioning its association with *Tamarix* and *Elaeagnus*, or on *Caragana* spp. as probably the sole plant genus of hosts for *A.halodendrihalodendri* (Pallas, 1773). Moreover, since the territory of this country is located between Eastern Europe and China, it may constitute a transit zone for the establishment of some quarantine pests from southern Asia. Therefore, it is extremely important to constantly examine and monitor, but also to preserve, these shrinking habitats.

The main threat to the unique habitats of Kazakhstan is the agricultural economy because approx. 81% of the total area of the country’s land is suitable for agriculture. Crucial changes in many ecosystems in Kazakhstan that are harmful to biodiversity occurred more than 50 years ago as a result of the extensive plowing of the steppe and forest-steppe zones. Together with the growth of livestock, a strong increase in overgrazing occurred in the region. As a result, there was a significant loss of biodiversity in the steppe areas. There are also several threats to the biodiversity of the desert habitats. Among them, the most important comprise the haphazard road network, the regulation of rivers and the illegal logging of saksaul. As a result of the urbanisation and intensive agricultural development in the foothills in the south and east of the country, the natural vegetation is still severely damaged. In the river valleys in the desert zone (e.g. the Syr Darya, Shu, Talas), due to the limitations of river flow, highly productive floodplain communities are almost completely degraded. Furthermore, the increasing pace of the construction of infrastructures such as roads, pipelines and power lines, creates a great negative impact on the fauna, even if only due to the fragmentation of habitats. In the last five years, the fields of oil and gas production and uranium mines have drastically been extended. The area occupied by mining enterprises is also expanding steadily. However, although many ecosystems, especially in the grasslands and abandoned pastures, began to be restored after the collapse of the USSR and the economic collapse in the 1990s and they have recently continued their natural recovery, previously abandoned areas are now being restored for the economic use. Unfortunately, the use of the current resource model of economic development leads not only to inefficient economic development but also to increasing pressure on ecosystems (MEWR 2014).

## Supplementary Material

XML Treatment for Psilotarsus brachypterus

XML Treatment for Psilotarsus brachypterus brachypterus

XML Treatment for Psilotarsus brachypterus pubiventris

XML Treatment for Anastrangalia sequensi

XML Treatment for Leptura annularis

XML Treatment for Leptura duodecimguttata

XML Treatment for Leptura quadrifasciata quadrifasciata

XML Treatment for Lepturalia nigripes rufipennis

XML Treatment for Lepturobosca virens

XML Treatment for Macroleptura thoracica

XML Treatment for Oedecnema gebleri

XML Treatment for Pachytodes erraticus

XML Treatment for Pseudovadonia livida bicarinata

XML Treatment for Stenurella bifasciata bifasciata

XML Treatment for Stenurella melanura melanura

XML Treatment for Strangalia attenuata

XML Treatment for Brachyta interrogationis russica

XML Treatment for Dinoptera collaris

XML Treatment for Stenocorus minutus

XML Treatment for Turanium scabrum

XML Treatment for Chlorophorus elaeagni

XML Treatment for Cyrtoclytus capra

XML Treatment for Echinocerus floralis

XML Treatment for Rhaphuma gracilipes

XML Treatment for Xylotrechus adspersus

XML Treatment for Xylotrechus alakolensis

XML Treatment for Xylotrechus capricornus

XML Treatment for Xylotrechus hircus

XML Treatment for Xylotrechus rusticus

XML Treatment for Trichoferus campestris

XML Treatment for Molorchus schmidti

XML Treatment for Obrium cantharinum cantharinum

XML Treatment for Amarysius duplicatus

XML Treatment for Anoplistes galusoi

XML Treatment for Anoplistes halodendri halodendri

XML Treatment for Anoplistes jacobsoni

XML Treatment for Cleroclytus semirufus collaris

XML Treatment for Aegomorphus clavipes

XML Treatment for Aegomorphus obscurior

XML Treatment for Agapanthia alternans alternans

XML Treatment for Agapanthia dahli calculensis

XML Treatment for Agapanthia villosoviridescens

XML Treatment for Agapanthia violacea

XML Treatment for Agapanthiola leucaspis

XML Treatment for Rhopaloscelis unifasciatus

XML Treatment for Dorcadion abakumovi sarkandicum

XML Treatment for Dorcadion absinthium ishkovi

XML Treatment for Dorcadion acutispinum

XML Treatment for Dorcadion arietinum arietinum

XML Treatment for Dorcadion arietinum charynense

XML Treatment for Dorcadion crassipes crassipes

XML Treatment for Dorcadion gebleri gebleri

XML Treatment for Dorcadion gebleri lukhtanovi

XML Treatment for Dorcadion kapchagaicum

XML Treatment for Dorcadion morozovi

XML Treatment for Dorcadion mystacinum rufidens

XML Treatment for Dorcadion nikolaevi

XML Treatment for Dorcadion semenovi semenovi

XML Treatment for Dorcadion sokolowi

XML Treatment for Dorcadion songaricum

XML Treatment for Dorcadion suvorovi konyrolenum

XML Treatment for Dorcadion tenuelineatum

XML Treatment for Dorcadion tianshanskii radkevitshi

XML Treatment for Dorcadion unidiscale

XML Treatment for Eodorcadion carinatum carinatum

XML Treatment for Politodorcadion eurygyne eurygyne

XML Treatment for Politodorcadion politum politum

XML Treatment for Politodorcadion ribbei bobrovi

XML Treatment for Exocentrus stierlini

XML Treatment for Mesosa myops

XML Treatment for Lamia textor

XML Treatment for Monochamus sartor urussovii

XML Treatment for Oberea kostini

XML Treatment for Oberea ruficeps ruficeps

XML Treatment for Phytoecia coerulescens

XML Treatment for Phytoecia rufipes rufipes

XML Treatment for Phytoecia nigricornis

XML Treatment for Menesia sulphurata

XML Treatment for Saperda alberti

XML Treatment for Saperda perforata

XML Treatment for Saperda scalaris

XML Treatment for Saperda similis
